# Supplementary material for: Investigating Scottish Long COVID community rehabilitation service models from the perspectives of people living with Long COVID and healthcare professionals: a qualitative descriptive study
Source: BMJ Open. 2023 Dec 14;13(12):e078740. doi: 10.1136/bmjopen-2023-078740 (PMC10729197; doi:10.1136/bmjopen-2023-078740)
Supplement: Supplementary data [file bmjopen-2023-078740supp002.pdf]

SUPPLEMENT File 1: Interview Topic Guides

Explanation of Supplement File 1
This file contains three interview topic guides, which were tailored for the three groups of study participants: 1) Long COVID leads; 2) Staff Delivering long COVID rehabilitation services (AHPs); and 3) People with long COVID (PwLC). The first guide, for long COVID leads, was informed by the Consolidated Framework for Implementation Research (CFIR). The CFIR categories are noted in blue.
Long COVID leads and AHPs were interviewed multiple times, whereas PwLC were interviewed at one time point. The second guide, for Staff Delivering long COVID rehabilitation, includes reframed questions in red for the second interview.

1. Interview Topic Guide for Long COVID Leads

Purpose

This topic guide supports the data collection to explore NHS Heath Board senior management perceptions of how decisions are made regarding the allocation of Scottish Government funding for Long COVID and in particular how decisions were/are made regarding funding, model of service provision and publicity for long COVID rehabilitation in community settings.

The topic guide is deliberately broad and more specific areas for questioning will be driven by the topics raised during the interview. You do not have to ask questions in the order they are presented but should use the questions as a checklist to ensure all key topic areas are covered.

SECTION 1: GENERAL INTRODUCTIONS

1.1 General introduction by interviewer to the purpose of the interview (Researcher note: relay purpose of interview as described above above).

1.2 General introduction by the interviewee of their managerial role in general and specifically in relation to long COVID.

1.2.1 Please could you tell me about your role within [name NHS Health Board]?

1.2.2 Specifically, could you tell me what role you have in the decision-making process regarding the Health Board's decision regarding delivery of long COVID? **(Process)**

1.2.3 If they do not mention their role in funding decisions relating to long COVID services probe for that.

SECTION 2: SERVICE DELIVERY

2.1 Can you describe to me how long COVID services have been provided to date by [name NHS Health Board]? (Service characteristics)

2.1.1 What are the services that are delivered for long COVID within [NHS Health Board]? (Service characteristics)

2.1.2 What were the drivers for introducing these services and the way in which they were/are delivered (internal {e.g. within Health Board}/external {political, societal etc})? **(Inner/Outer setting; Process)**

2.1.1 How were these services funded? **(Inner/Outer settings)**

2.1.2 Have long COVID services changed at any point within your service? [If yes, then probe regarding why they changed and in what way]. **(Service characteristics/ Inner/Outer setting)**

2.1.2 What are/were the challenges that [NHS Health Board] face(d) in delivering long COVID services? **(Inner/Outer settings; Process)**

2.3 How have your services changed (if at all) since the arrival of COVID?

2.3.1 In what way/if any/ has [NHS Health Board] promoted these services to key referrer agencies (such as GPs and others in Primary Care), and the public? What has guided the decisions you have made re promoting long COVID services to Primary Care, the community and other stakeholders?

2.3. 1 What is your general attitude towards this model of community rehabilitation? (How has it been going)? [Prompts: positive/beneficial – please elaborate, negative/detrimental – please elaborate, unclear etc.]

2.2 On 30 September 2021 the Scottish Government announced additional funding of £10 million pounds for long COVID services. How has that allocation been decided for NHS Health Boards? What share did [NHS Health Board] receive? (Inner/Outer settings; Processes)

2.2.1 Within [NHS Health Board] what was the decision-making process for how that money would be spent? **(Inner setting; Processes)**

2.2.2. Will rehabilitation services for people with long COVID in the community benefit from this funding? If yes, then how? If no, then why was spending not priorities for this? **(Intervention; Processes; Inner setting)**

2.2.3 If there will be new services, how will these new services be communicated to Primary Care and the public and other relevant stakeholders?

SECTION 3: FACILITATORS AND BARRIERS

3.1 What (if anything) do you think is working well about how long COVID services – and particularly rehabilitation for people with long COVID are being delivered for people in [NHS Health Board].

3.2.1 What has enabled this/these to work well?

3.2 What (if any) barriers do you feel there are in delivering high quality services for people with long COVID, in particular that requiring long COVID rehabilitation? How might these barriers be overcome?

Duncan E, *et al. BMJ Open* 2023; 13:e078740. doi: 10.1136/bmjopen-2023-078740

SECTION 4: THE FUTURE

4.2 How do you see long COVID services being delivered over the coming months and years?
(Intervention; Outer/Inner Setting; Process)

4.1 How do you foresee long COVID rehabilitation in [NHS Health Board] being delivered over the coming months and years?

4.1.1 What are the funding implications?

SECTION 5: CLOSING THE INTERVIEW

5.1 Is there anything else we have not covered regarding funding and delivery of long COVID, particularly in relation to rehabilitation in the community?

Notes for interviewer

- Check topic guide and return to any questions as necessary
- If no further questions/discussion remains, thank participant for their time
- Save/stop the recording
- Note down any interviewer/participant observations
- Upload recording to CSO R Drive (RGU) and include any notes taken

Duncan E, *et al. BMJ Open* 2023; 13:e078740. doi: 10.1136/bmjopen-2023-078740

2. Interview Topic Guide for Staff Delivering Long COVID Rehabilitation Services (AHPs)

Aim

To explore staff perceptions of how community rehabilitation for people experiencing symptoms of long COVID is being delivered. To explore their perception of the barriers (and how these could be overcome) and facilitators (and how these could be optimised) for success. To explore their perception of the impact of community rehabilitation on patient outcomes for this population. The interviews have a general purpose to obtain feedback from those involved in delivering community rehabilitation for people with long COVID.

The specific aim of follow-up interviews is to get a sense of what, if anything has changed in the last few months. This could be at an organisational level, or at an individual professional level in relation to long COVID.

Remember – we want you to have a good conversation with people about community rehabilitation for people with long COVID. The questions are there to provide a bit of structure and to make sure you cover all required basis, but it is not an interview schedule and you do not have to follow them in order.

Tasks at or before start of interview:

(Re)Introduce self and relax participant through welcoming conversation

Check participant has read participant information sheet and understood aims and consents

Assign code to each new participant (e.g. L001 for Lanarkshire participant). Use previous code if a repeat interview assigning the letter b (if 2nd interview).

Participant information to be gathered at start of each interview: profession, gender, years qualified, Agenda for Change band (Ask if what 'Band' they are; this is their professional grade), length of time they have worked in community rehabilitation. Check that details have stayed the same since last interview if it is a repeat interview. If not, note new details.

SECTION 1: GETTING STARTED

Can you describe your role within the community rehabilitation team?

How has this role been for the last few months? Have there been any changes in relation to long COVID patients?

In this role, have you had any seen any/many patients who are experiencing long COVID?

> *(if they have seen long COVID patients)* Can you tell me about what your role has been with patients with long COVID? Has this changed at all over the last few months?
>
> *(if they have seen long COVID patients)* How confident have you been in delivering therapy for people with long COVID symptoms? Has this changed at all over the last few months?

(if not seen any) Have you been aware of any of your colleagues treating people due to problems related to long COVID? What have they done? Has this changed at all over the last few months?

SECTION 2: SERVICE DELIVERY

(Covers TiDIER criteria – Why, What Who, How, Where, When & How much, Tailoring)

Tell me about rehabilitation for patients with long COVID within your team/department?

Have there been any changes to how long COVID is delivered in your service? If so, can you describe these changes?

- How are people referred to your service? Who by? How do you let people know about your service?
- Why are people being referred for long COVID rehabilitation?
  - What problems? What are the general goals of rehabilitation for the people that have been seen?
- What happens after you received a referral (triage? Who decides who sees the person? How is initial contact (telephone/online/f2f)
- What happens after initial contact?
- What rehab services are offered?
  - e.g. assessment & advice only/self-management & signposting/set number of sessions/individual to patient needs.
  - How is that process decided?
  - Who provides rehabilitation? How is it decided who sees each patient?
- Where forms of therapy are generally delivered?
  - what do the professionals do?
- Typically, how intense is the rehabilitation process?
  - frequency/duration of interventions, how long on caseload?
- Is this different or the same as other patients accessing your service/department?
- In what way is the therapy tailored to the individual being treated?
- How do you decide when to stop intervention?

SECTION 3: FACILITATORS AND BARRIERS

What is your perception of the potential of community rehabilitation for people with long COVID?

Has this changed at all over the last few months?

- What seems to work well for people who have long COVID? (Consider: engagement, outcomes, access to services, development of service etc.)
  - Why/in what way?

- What doesn't work so well in delivering community rehabilitation for people with long COVID (Consider: referral process, mode of therapy delivery, duration of intervention, admin, resources etc.)
    - Why/how could these be overcome?

SECTION 4: THE FUTURE

How could services change to better suit needs of patients with long COVID who need rehabilitation?

What do you see as the big challenges facing long COVID rehabilitation just now?

- Consider: staffing: funding, mode of delivery, any services not currently provided, referral to other services, central long COVID clinics vs integrated services within existing structures.

SECTION 5: CLOSING THE INTERVIEW

Is there anything else we have not talked about but you think would be beneficial in our understanding of community rehabilitation for people with long COVID?

Notes for interviewer:

- Check topic guide that all key areas are covered
- Thank participant for their time
- Save and stop recording
- Note down any participant observations or thoughts re the interview
- Upload recording to CSOR Drive (RGU) with any notes taken

Duncan E, *et al. BMJ Open* 2023; 13:e078740. doi: 10.1136/bmjopen-2023-078740

3. Interview Guide for People with Long COVID (PwLC)

Aim

To obtain input from people living with long COVID, their perceptions of the acceptability of the community rehabilitation service, their preferences for service delivery models and what a responsive service might look like (referral, location, duration, modes of delivery etc…).

Interviews have a general purpose to obtain ideas from participants about what an ideal community rehabilitation service for people living with long COVID might look like, what they perceive to be the barriers and facilitators to the model they are experiencing and what gaps, if any, they feel exist in service delivery. Therefore, the topic guide is deliberately broad and more specific areas for questioning will be driven by the questions and interests of the people taking part.

Welcome and introductions.

Check they can hear ok.

Thank you for agreeing to meet with me. Introduce self to participant.

I hopefully won't take too much your time up.

Structure of call.

Could I just start by going though the verbal consent, just to make sure that you are happy with everything? Inform them that we will go through the consent process which asks a few statements and if you can say yes/no that would be great. Just so you know that the interview will be audio recorded just so we can refer back to the data if that's ok with you.

I have 8 statements that I will read and if you are happy, if you can just say yes at the end of them that would be great.

Ask to say name. <CONSENT>

Great, thank you for doing that – it just keeps us all right. I will stop the recording there.

Opportunities for the participant to ask questions about the study and to confirm verbally that they are still happy to participate.

Re-start new recording

Opening statement:

Thank you again for taking the time to speak to me. And just to give you a bit of background about the study. Essentially, we're doing a project over two years just looking at long COVID rehabilitation across Scotland. We're looking across four Scottish health boards. And as you can understand we are still learning about long COVID and things are changing very quickly. So, we are kind of trying to see how things are changing over these two years right from start in relations to rehabilitation service. What we've been doing is speaking to staff that deliver the service across Scotland also sort of managers and directors at each of those health boards. But we also want to hear from people like yourselves and who have experienced COVID and experiencing long COVID, just in terms of getting

your viewpoints about your experiences, what's been good, what's been bad and so forth. We are really interested to hear things from your side of things if that's alright?

Start Interview questions

These can be asked in any order and are there to guide discussion. Feel free to use your own words to ask the questions in as open a manner as possible. What is important is the topics are covered.

Q1. Can you tell me a bit about your COVID story?

Prompts: When were you diagnosed? Was it a formal diagnosis following a test or did you feel you matched the symptoms? When did you begin to consider you had long COVID? How is COVID affecting you now?

Prompts:
Job: What is your job now?
Symptoms mentioned? Any other symptoms?
Did you seek any help from GP? What did the GP do? Refer to other services? What services?
And how did that work for you?
Has that changed? Have you had any face-to-face appointments or is it just stayed virtual, online?
Anything you thought could be better? What do you think went well for you?

Q2. How did you get referred for long COVID rehabilitation?

Prompts: Who referred you?
What did they refer you for (e.g. fatigue, breathlessness, brain-fog etc.),
was it easy to get referred?
How long did they wait to be seen?

Q3. For how long and how often have you been receiving long COVID rehabilitation (weeks and sessions [**when and how much]**)?

Q4. What had you hoped/do you hope to achieve from community rehabilitation?

Q5. Can you tell me a bit about the rehabilitation you have received to date?

Prompts: **Who** has provided your long COVID rehab (i.e. what type of professionals – physiotherapists, occupational therapists etc);
What has your rehabilitation consisted of so far (i.e. what procedures or advice, or other activities); **How** was it provided? (e.g. face to face or in a group, online or telephone);
Where was it provided? (if face to face was it at home or in a clinic etc)

Q6. What are your experiences of long COVID rehabilitation to date?

Prompts: Do you feel the service is/was responsive to your needs?
How do you feel about the number of times and frequency with which you were seen?
How appropriate do you think the service's way of delivering rehabilitation has been/is (e.g. face to face/digital/self-management) for you?

Q7. Are there any particular aspects that you think are working well or lessons learned to improve long COVID rehabilitation you are receiving?

Prompt: What is working well?
What could be better? (Note – try and tap into service issues such as referral, delivery mode etc, as well as individual issues)

Q8. Is there anything else you would like to share about your experience of the community rehabilitation service?

Closing statement:

That's all the questions I've got. Is there anything else that we've not touched on that you think you know important to mention.

Great. Well, thank you again. Lovely to meet you and thank you. As you said, you're happy enough if there's anything else in the future, if you're happy for me to get back in touch.

I was going to ask if there's anything in the future that I think of, would you be happy for me to contact you again just via email or something and see how you're getting on?

Thanks, and conclude interview.

Turn off recorder

Duncan E, *et al. BMJ Open* 2023; 13:e078740. doi: 10.1136/bmjopen-2023-078740
