# Supplementary material for: Investigating Scottish Long COVID community rehabilitation service models from the perspectives of people living with Long COVID and healthcare professionals: a qualitative descriptive study
Source: BMJ Open. 2023 Dec 14;13(12):e078740. doi: 10.1136/bmjopen-2023-078740 (PMC10729197; doi:10.1136/bmjopen-2023-078740)
Supplement: Author's manuscript [file bmjopen-2023-078740.draft_revisions.pdf]

BMJ Open

BMJ Open is committed to open peer review. As part of this commitment we make the peer review history of every article we publish publicly available.

When an article is published we post the peer reviewers' comments and the authors' responses online. We also post the versions of the paper that were used during peer review. These are the versions that the peer review comments apply to.

The versions of the paper that follow are the versions that were submitted during the peer review process. They are not the versions of record or the final published versions. They should not be cited or distributed as the published version of this manuscript.

BMJ Open is an open access journal and the full, final, typeset and author-corrected version of record of the manuscript is available on our site with no access controls, subscription charges or pay-per-view fees (http://bmjopen.bmj.com).

If you have any questions on BMJ Open's open peer review process please email info.bmjopen@bmj.com

BMJ Open

Perceptions and experiences of different Long COVID community rehabilitation service models from the perspectives of people living with Long COVID and healthcare professionals

Journal:	*BMJ Open*
Manuscript ID	bmjopen-2023-078740
Article Type:	Original research
Date Submitted by the Author:	10-Aug-2023
Complete List of Authors:	Duncan, Edward; University of Stirling, NMAHP-RU<br>Alexander, L; Scottish Centre for Evidence-Based Multi-Professional Practice; Robert Gordon University, School of Health Sciences<br>Cowie, Julie; Glasgow Caledonian University, NMAHP Research Unit<br>Morris, Jacqui H.; University of Dundee, SNHS<br>Moss, Rachel; Healthcare Improvement Scotland<br>Preston, Jenny; Ayrshire Central Hospital, Douglas Grant Rehabilitation Centre<br>Shim, Joanna; Robert Gordon University, School of Health Sciences<br>Stage, Emma; Robert Gordon University, School of Health Sciences<br>Tooman, Tricia; University of Stirling, NMAHP-RU<br>Cooper, K; Robert Gordon University, School of Health Sciences
Keywords:	Post-Acute COVID-19 Syndrome, HEALTH SERVICES ADMINISTRATION & MANAGEMENT, REHABILITATION MEDICINE

SCHOLARONE™
Manuscripts

I, the Submitting Author has the right to grant and does grant on behalf of all authors of the Work (as defined in the below author licence), an exclusive licence and/or a non-exclusive licence for contributions from authors who are: i) UK Crown employees; ii) where BMJ has agreed a CC-BY licence shall apply, and/or iii) in accordance with the terms applicable for US Federal Government officers or employees acting as part of their official duties; on a worldwide, perpetual, irrevocable, royalty-free basis to BMJ Publishing Group Ltd ("BMJ") its licensees and where the relevant Journal is co-owned by BMJ to the co-owners of the Journal, to publish the Work in this journal and any other BMJ products and to exploit all rights, as set out in our licence.

The Submitting Author accepts and understands that any supply made under these terms is made by BMJ to the Submitting Author unless you are acting as an employee on behalf of your employer or a postgraduate student of an affiliated institution which is paying any applicable article publishing charge ("APC") for Open Access articles. Where the Submitting Author wishes to make the Work available on an Open Access basis (and intends to pay the relevant APC), the terms of reuse of such Open Access shall be governed by a Creative Commons licence – details of these licences and which Creative Commons licence will apply to this Work are set out in our licence referred to above.

Other than as permitted in any relevant BMJ Author's Self Archiving Policies, I confirm this Work has not been accepted for publication elsewhere, is not being considered for publication elsewhere and does not duplicate material already published. I confirm all authors consent to publication of this Work and authorise the granting of this licence.

For peer review only

TITLE: Perceptions and experiences of different Long COVID community rehabilitation service models from the perspectives of people living with Long COVID and healthcare professionals

ABSTRACT

Objectives: To explore the perceptions and experiences of barriers and facilitators to accessing Long COVID community rehabilitation.

Design: We used a qualitative descriptive design over two rounds of data collection with three participant groups: i) people with experience of rehabilitation for Long COVID (PwLC), ii) NHS staff delivering and/or managing community rehabilitation services (allied health professionals (AHPs)), and iii) NHS staff involved in strategic planning around Long COVID in their health board (Long COVID leads).

Setting: Four NHS Scotland territorial health boards.

Participants: 51 interviews: eight Long COVID leads (11 interviews), 15 AHPs (25 interviews), and 15 PwLC (15 interviews).

Results: Three key themes were identified: i) Accessing care for PwLC, ii) Understanding Long COVID and its management, and iii) Strengths and limitations of existing Long COVID rehabilitation services.

Conclusions: Organisational delivery of Long COVID community rehabilitation is complex and presents multiple challenges. In addition, access to Long COVID community rehabilitation can be challenging. When accessed, these services are valued by PwLC but require adequate planning, publicity, and resource. The findings presented here can be used by those developing and delivering services for people with Long COVID.

Strengths and limitations of this study

- This is the first study to explore perceptions and experiences of different community rehabilitation models for Long COVID in the Scottish context
- Perspectives of people with Long COVID, staff delivering services, and staff leading on Long COVID in four geographically diverse Scottish health boards are included
- The strengths and limitations of different Long COVID rehabilitation service model components are highlighted
- The rapidly evolving nature of Long COVID and its management resulted in fewer distinct differences between the four health boards than originally anticipated

For peer review only - http://bmjopen.bmj.com/site/about/guidelines.xhtml

INTRODUCTION

Background

Long COVID, a condition defined as ongoing COVID-19 symptoms that continue beyond twelve weeks following an initial acute COVID-19 infection, was first described in Spring 2020 [1]. It is estimated that at least 65 million people globally have Long Covid [2]. By January 2023, over 2 million people were estimated to have Long COVID in the United Kingdom (UK), including over 175,000 living in Scotland [3]. Long COVID has broad multiple system presentation and can have profound physical, emotional, social, and financial consequences. Moreover, debilitating symptoms of Long COVID often persist for a year or more [3].

NHS funding to support the diagnosis, treatment, and rehabilitation for Long COVID varies across the UK. Since the autumn of 2020, NHS England have allocated £94 million (£1.66 per capita, based on the 2021 UK Office of National Statistics Census data England's population was 56,489,800) to be invested in specialist Long COVID clinics in England to complement existing primary, community, and rehabilitation care services [4]. The Welsh Government has allocated £18.3 million since 2020 (£5.88 per capita based on a population of 3,107,500 in 2021) to support the delivery of Long COVID diagnosis, treatment, and rehabilitation within existing services [5]. In September 2021, the Scottish Government announced £10 million (£1.82 per capita based on a population of 5,479,900 in 2021) over 3 years to support Scottish NHS health boards' response to Long COVID [6]. At the end of May 2022, plans for the first £3 million of that funding were announced.

The National Institute for Health and Care Excellence (NICE) [1] and The World Health Organisation [7] recommend people with Long COVID, following an initial medical assessment to exclude underlying conditions, have access to rehabilitation to aid their recovery. As with other long-term conditions, community rehabilitation for people with Long COVID should be personalised, multidisciplinary, and comprehensive in order to maximise function, quality of life, and participation in society [8 9].

However, the optimal approach to deliver Long COVID community rehabilitation is currently unknown. NICE clinical guidance suggests broad rehabilitation approaches, which can be delivered in isolation or in combination, such as self-management and multidisciplinary rehabilitation [1]. The effectiveness of these approaches is not yet clear and there are further uncertainties about the most appropriate means of organising service delivery, particularly that of multidisciplinary rehabilitation. Furthermore, there are well-documented barriers to accessing healthcare services for PwLC [10-12]. Our 2019 national survey [13] of Scotland's 14 territorial health boards found

For peer review only - http://bmjopen.bmj.com/site/about/guidelines.xhtml

that Long COVID community rehabilitation was universally available across Scotland. At the time of the survey, one health board was delivering Long COVID community rehabilitation as a dedicated service, with the remainder integrating rehabilitation within existing services. And yet, there was substantial variation across health boards in the mode of service delivery, given that the means of optimally delivering community rehabilitation to this population was unknown.

To date rehabilitation services for Long COVID have varied internationally with respect to delivery and the healthcare professionals involved [14], including virtual rehabilitation focusing on self-management and education [15], group-based pulmonary rehabilitation [16], tiered multidisciplinary pathways [17], and phased approaches to managing symptoms [18]. Our research group are investigating models of service delivery for Long COVID rehabilitation and will provide recommendations for the effective provision of community rehabilitation for PwLC. As part of our larger study, we are exploring the experiences and perceptions of people receiving rehabilitation for Long COVID and NHS staff (operational and strategic) involved in delivering or managing Long COVID community rehabilitation. The aim of this phase of the study was to explore the perceptions and experiences of barriers and facilitators to Long COVID community rehabilitation as services evolved over a 12-month period.

METHODS

Study Design

This paper reports on the first two rounds of data collection of a longitudinal qualitative descriptive [19] study based on 51 interviews with three participant groups; i) people with experience of rehabilitation for Long COVID (PwLC); ii) NHS staff delivering and/or managing community rehabilitation services (allied health professionals (AHPs)), and iii) NHS staff involved in strategic planning around Long COVID in their health board (Long COVID leads). The study is reported in accordance with the Consolidated criteria for reporting qualitative research [COREQ] [20].

Setting

The study was conducted in four Scottish health boards (HB1-HB4). The boards were selected for their geographical and demographic spread as well as variation in Long COVID rehabilitation service models, summarised in Table 1. As shown in Table 1, all boards adopted some form of multi-disciplinary team (MDT) approach using a blended mode of delivery (face-to-face, telephone, Near Me). Staffing varied; occupational therapy and physiotherapy were most consistent across services, with some including additional professions such as dietetics, speech and language therapy, and psychology. Notably, none of the services included medical staff.

For peer review only - http://bmjopen.bmj.com/site/about/guidelines.xhtml

Table 1: Description of participating health boards

Health Board	Health Board 1	Health Board 2	Health Board 3	Health Board 4
**Setting**	Largely rural	Large urban/rural area	Largely urban	Large urban/rural area
**Round 1 Long COVID service delivery model**	Integrated service: community rapid response rehabilitation	Integrated service: community rehabilitation	Integrated service: community rehabilitation	Dedicated long COVID service
	**MDT:** Physiotherapy, Occupational Therapy, Nursing, Community Psychiatric Nurse (CPN), Pharmacy, Advanced Nurse Practitioner (ANP), and Speech and Language Therapy, with links to GP and Dietetics	**MDT:** Physiotherapy, Occupational Therapy, Dietetics, and Speech and Language Therapy.	**MDT:** Physiotherapy, Occupational Therapy, Psychology, Speech and Language Therapy, and Rehabilitation Assistant Practitioner, Physiotherapy, Occupational Therapy, Psychology, Dietetics, and Speech and Language Therapy	**MDT:** Physiotherapy, Occupational Therapy, Psychology, Dietetics, and Speech and Language Therapy
	Blended delivery	Blended delivery with centralised point of referral	Blended delivery	Blended delivery
**Round 2 Long COVID service delivery model**	As Round 1	As Round 1	Integrated service moving towards a soft launch of dedicated long COVID Service<br><br>Blended delivery with single access point	Dedicated Long COVID Service via a 3-tiered triage system<br><br>Service temporarily halted due to large volume of referrals and subsequent decline in staffing and funding

Key: MDT=Multidisciplinary

For peer review only - http://bmjopen.bmj.com/site/about/guidelines.xhtml

Recruitment and Data Collection

Two rounds of data collection took place: Round 1 (November 2021 – April 2022) and Round 2 (June – July 2022). In each round, three participant groups took part: PwLC, AHPs, and Long COVID leads. Long COVID leads were purposively sampled by email invitation or word of mouth, for their role in leading on Long COVID within their health board, and included AHP directors, medical directors, and clinical service leads. AHPs, most often occupational therapists and physiotherapists, were involved in providing Long COVID rehabilitation. Most AHPs acted in a team member role, though a small number were also clinical service leads. AHPs involved in the treatment of PwLC in the study health board areas were purposively recruited by email invitation. The invitation was sent by senior managers on behalf of the study team and explained the reasons for the research. Finally, a convenience sample of PwLC were recruited by their AHP. PwLC were provided with a study pack in printed or electronic format that explained the purpose of the study and volunteers opted into the study by contacting the research team by email, telephone, or leaving their contact details after completing a survey of health-related outcomes as part of the larger study.

Interview topic guides [see Supplemental file 1] were developed by the research team that included people with lived experience of Long COVID. Topic guides were further refined for Round 2 to take account of the temporal nature of the questions being asked. It combined a theory-based approach using the TIDieR intervention description categories [21], and a series of questions exploring facilitators and barriers to delivering and receiving community Long COVID rehabilitation. The interview topic guide was pilot tested with people who had lived experienced of Long Covid and with two AHPs who were not involved in the study prior to implementation. Web-based [Microsoft Teams] or telephone interviews were conducted by five experienced healthcare researchers with master's or doctoral level qualifications and experience of qualitative research [VB, JC, RM, JS, TT]. All interviewers were female and had experience of qualitative NHS data collection and analysis. Two of the researchers were AHPs and had prior awareness of the challenges of delivering rehabilitation. All interviewers were provided with training and supervision from KC and ED and had no prior relationship with the services or participants. Informed consent was obtained from all participants and recorded verbally prior to each interview. Interviews lasted between 22 and 86 minutes (average 52 minutes). Only the participant and interviewer were present during each interview. Interviews were audio recorded and transcribed intelligently by an external transcription service, where redundant words and/or sounds were removed. Field notes were taken during some interviews. These notes added to team discussions to support understanding of the data as it was collected but did not form part of the official data analysis. Transcripts were not returned to participants for comment or correction.

For peer review only - http://bmjopen.bmj.com/site/about/guidelines.xhtml

Data analysis

Data were analysed using the Framework method, which is commonly used in applied health research and recognised as appropriate for multidisciplinary research teams [22].

For each participant group, at least two researchers from the team familiarised themselves with at least 20% of transcripts, making analytical notes directly on the transcripts. Following discussion between all team members involved in familiarisation, a framework for analysis and interpretation was constructed [23]. Although line-by-line coding is common, it is also possible to develop a framework without engaging in explicit coding [23]; we adopted the latter approach. The 'analytical framework' [22] was applied across all interview transcripts using highlighting and the comments function in Microsoft Word, with regular review and discussion within the team. A charting matrix was created in Microsoft Excel that involved summarising broad categories of data from the transcripts, then interpreting the charted data by exploring within and between-cases to derive concepts and themes. These were then grouped into overarching themes. All researchers were involved in interpreting the data during regular team discussions. Participants did not directly provide feedback on the findings. However, the findings were presented in a webinar attended by Long Covid Leads, AHPs and PwLC, several of whom had participated in the study. The webinar attendees endorsed the study findings and reflected that they resonated with their personal experiences.

Patient and Public Involvement

Two people with lived experience of Long COVID were core members of the research team. They co-developed study materials and interview topic guides and contributed to analysis and interpretation of study findings.

RESULTS

Participant characteristics

We recruited eight Long COVID leads, who took part in 11 interviews over the two rounds of data collection: 15 AHPs (25 interviews) and 15 PwLC (15 interviews). See Table 2 for participant details. No participants who consented withdrew from the study.

For peer review only

For peer review only - http://bmjopen.bmj.com/site/about/guidelines.xhtml

Table 2 Participant demographics

	Long COVID leads (L)	Staff delivering service/AHPs (S)	Patients (P)
	**N=6**<br>**Rounds 1 & 2 N=3**<br>**Round 1 N=2**<br>**Round 2 N=1**	**N=15**<br>**Rounds 1 & 2 N=9**<br>**Round 1 N=2**<br>**Round 2 N=4**	**N=15**
**Age:**			
Working age [%]	8 [100]	15 [100]	13 [87]
		AfC Band / **N=**	
		6 / 7	
		7 / 4	
		8a / 4	
**Sex:**			
Female [%]	6 [75]	15 [100]	12 [80]
Male [%]	2 [25]	0	3 [20]
**Employment status:**			
Working full-time [%]	8 [100]	15 [100]	6 [40]
Working part-time [%]	0	0	1 [7]
Phased return to work [%]	0	0	2 [13]
Retired [%]	0	0	2 [13]
Unable to work because of illness [%]	0	0	3 [20]
Not provided [%]	0	0	1 [7]
**Time from first COVID diagnosis**	NA	NA	12 – 28 months

Key: AHPs = Allied Health Professionals; AfC = Agenda for Change

For peer review only

Findings

Framework analysis resulted in three key themes being identified: i) Accessing care for PwLC, ii) Understanding Long COVID and its management; and iii) Strengths and limitations of existing long COVID rehabilitation services. Table 3 illustrates the three themes and the categories of data that contributed to each.

Table 3: Key themes and categories of data

Key Themes	Sub themes
**Accessing care for people with Long COVID**	PwLC accessing General Practitioner (GP) services [L; P]<br>GP referring PwLC to community rehabilitation [L; P; S]<br>Awareness of services by GPs & PwLC [S]<br>Signposting [S]<br>Waiting lists & waiting times [P]
**Understanding Long COVID and its management**	Long COVID prevalence [L]<br>Long COVID unknowns [L; P]<br>Reluctance to diagnose [P]<br>Tests/investigations [P]<br>Being believed [P]<br>Evolving evidence-base [L; S]<br>Education of PwLC and community rehabilitation staff [S]<br>Information sharing [S]<br>Learning from other conditions [S]<br>Confidence of community rehabilitation staff [S]
**Long COVID services**	Current services strengths & limitations [L; P; S]<br>Resourcing services [L; P; S]<br>Need for Long COVID services [L; P; S]<br>Navigating politics [L]

Key: L = Long Covid leads; P = PwLC; S = AHPs

Participant data was coded and reported as follows: (1) Role of participant: L = Long Covid leads; P = PwLC; S = AHPs; (2) Health Board (HB) number: HBs 1-4; (3) Round of data collection: 1 or 2; and (4) Participant number (e.g. the participant identifier for the first quote: P-3-2-08 is a PwLC from HB3, data was collected in Round 2 and was the 8th PwLC interviewed).

For peer review only - http://bmjopen.bmj.com/site/about/guidelines.xhtml

I: Accessing care for People with Long COVID

Barriers to accessing Long COVID rehabilitation were identified by all participant groups. In Round 1, patients commonly attributed this to limited availability of face-to-face GP appointments due to COVID-19 restrictions.

> *"You don't get face-to-face with the GP. So again, when you get a GP, it's always a different GP you're talking to and you try your best, but you get locum, and they've all been fantastic. I'm not, again, I wouldn't criticise them, but it's exhausting telling the same story every single time." [P3208]*

Long COVID leads and AHPs observed people's reluctance to seek help from services widely acknowledged as being under pressure. By Round 2, they felt that the perception of GPs being closed, and services overwhelmed, needed to be questioned, and called for greater public awareness of Long COVID rehabilitation services and how to access them.

> *"[We] need to get past the public's perception that GPs are shut so they return to their GP and get a referral to their service if required." [L3102]*

All participant groups were aware of the dedicated Long COVID service in HB4. However, due to the publicity the service had received and the pro-active development of a clear GP referral pathway, demand for this service quickly exceeded capacity. Staff (AHPs and Long COVID leads) expressed concern that promoting a Long COVID service in their own board may result in the same situation. Their concerns were compounded by the widely acknowledged pressure in the healthcare system due to a range of factors including staff redeployment, absence, and reopening of non-essential rehabilitation services that had been closed during previous phases of the pandemic.

> *"They* [HB4 Long COVID service] *were basically inundated. So, I had to wait, about four months or so, something like that for a slot. But it was quicker than I thought, but they were just getting overwhelmed with the workload." [P4101*]

> *"Yeah. So, it's [integrated service] not a widely publicised thing, because I don't know if we could cope as a service with the numbers." [S1102]*

For peer review only - http://bmjopen.bmj.com/site/about/guidelines.xhtml

Patients and staff reported both frustration and understanding around long waiting times for Long COVID rehabilitation:

> *"The referral side of it they probably done all the right things, but the timescales were just probably horrific but understandable. I know that in hindsight now so, but it didn't take away the frustrations of it." [P3208]*

> *"Difficult due to capacity. Everywhere so busy and up against it. The pressures on us are really extreme." [L3101]*

The dedicated service in HB4 was accessed by self-referral and delivered by AHP staff. Self-referral was viewed positively by patients; however, staff reported that the lack of information on self-referrals made appropriate triage of patients challenging. Health boards without self-referral and/or a Long COVID service also expressed concern that people with Long COVID would not be prioritised for rehabilitation within usual community rehabilitation services.

> *"I don't have self-referral at the moment. And the referrals coming through the GP or through something, some of them are coming through. But I don't know where all the others are, so, I'm not blocking access, but they are getting stuck in the system, in my system, in that if I don't have enough information on the referral, they're just going on to the normal routine waiting list." [S2207]*

HB4 reported the lack of medical staff as a limitation to their dedicated service and, in keeping with other health boards, reported that the lack of a clear Long COVID pathway was a limitation, particularly as people often presented with complex issues that were unsuitable for immediate rehabilitation.

> *"But it's those that have the more complex needs that I wouldn't know where to send them to." [S2103]*

II: Understanding Long COVID and its management

Patients commented on the burdensome nature of managing Long COVID and there being a reluctance among GPs to provide a diagnosis. They noted concerns about documenting Long COVID in healthcare records and a need to rule out other underlying conditions.

For peer review only - http://bmjopen.bmj.com/site/about/guidelines.xhtml

"It's quite difficult and just feel like, you know, you have a list as long as your arm when you actually speak to the GP because some things have changed and you know they're very sympathetic, but very clear and honest that well, we don't really know, you know? We just don't know. So, we'll give you this pill to try and treat this symptom at the moment and, that's quite hard.' [P4101]

The lack of diagnosis was also observed by AHPs and Long COVID leads, who shared a discomfort associated with labelling patients.

"What are we shaming people for having a condition? What's that all about? It's very bizarre. You get them help. I don't understand. It's like invisible illnesses." [L1202]

"It was really difficult actually to know whether to adopt the term Long COVID 'cause it's not a medical term, but we've decided in the end it's probably got wider recognition, now, that's what the patients are using. But having to be very careful, actually even when you're writing letters to GPs, you know if they didn't have confirmed COVID in the beginning, it's a presumed COVID illness, you know symptoms consistent with Long COVID, you know. It's a lot of kind of working around the houses I feel." [S4101]

Most patients expressed a need to be heard and believed by healthcare professionals. They also felt that there was powerful value in hearing that other people with Long COVID experienced similar symptoms, which provided a helpful process in validating patients' unmet needs.

"It felt to me like it was a bunch of very random symptoms. It didn't seem connected, and I think probably one of the best things in that first conversation was just hearing that the symptoms I have were very common and they are all related." [P4102]

"I get the impression that a lot of my symptoms are replicated a lot across, across other people so and I'm getting a lot of assurance on what people's progress like recovery-wise again its always pinned by keep your expectations to a minimum, like it's no [not] *going to be the miracle cure." [P3208]*

Both AHPs and Long COVID leads spoke of the challenges of understanding Long COVID and its constantly evolving evidence base, as well as the lack of data on prevalence. AHPs also reported a

For peer review only - http://bmjopen.bmj.com/site/about/guidelines.xhtml

lack of evidence to support best practice for managing Long COVID, which could result in anxiety around patient management.

> *"So, what I was concerned about was what if I give the wrong advice or the wrong sort of exercise prescription, and that I actually cause harm to him by something that I've done. And I watched some of the podcasts to sort of understand what we should and shouldn't be doing, and that is when I started to realize that there is a really strong link with Long COVID and the chronic fatigue syndrome and ME population who have been saying for years and years that, actually, sometimes, graded exercise therapy is really harmful, and you absolutely should not prescribe it." [S2104]*

To increase understanding of Long COVID, AHPs sought out peer support, online resources, and knowledge from other conditions to determine how to support people with Long COVID.

> *"There's certainly discussions around what is the best way to approach management of Long COVID and there's been a few things we can do, though, because nationally there's a huge focus on physiotherapy management of Long COVID you know, the CSP [Chartered Society of Physiotherapists], I've got lots of stuff out there, but we've also got the clinicians and the COVID rehab team there that although they're not seeing patients and more than happy to signpost people to resources and help with a bit of professional advice. I would say from a Community hub, clinician point of view, confidence hasn't been the main issue. They do seek professional advice on how to manage them." [S3203]*

III: Strengths and limitations of existing Long COVID services

A characteristic of all participating health boards during the study period was the dynamically evolving nature of the services provided. This was partly due to external factors relating to COVID prevalence, the restrictions this placed on service delivery, and financial planning decisions. Two health board areas changed their organisational structures affecting services. One health board (HB4) commenced a limited dedicated Long COVID rehabilitation service, delivered by an occupational therapist and a physiotherapist. The service developed clear pathways for referral that gained considerable publicity, as a result of which, the service was unable to meet demand. This was compounded by staff leaving their post. A considerable waiting list developed. The service ran for 18 months after which there was no further funding to continue its delivery, the service ceased and individuals on the waiting list were distributed to local community rehabilitation teams.

For peer review only - http://bmjopen.bmj.com/site/about/guidelines.xhtml

Strengths

By Round 2 of data collection, AHPs in all health boards were able to see PwLC face-to-face, conduct home visits, have greater integration into the community, and begin working towards return-to-work packages with appropriate Long COVID patients. All these service features were perceived by clinicians to increase the flexibility of service delivery. Patients had experienced a range of modes of service delivery, largely a blended model of face-to-face individual, group, or online interventions. However, irrespective of the delivery model, most patients had a positive experience of Long COVID rehabilitation, especially the self-management information they received. PwLC reported that validation of their experience was particularly significant.

> *"I'm certainly more educated, already daily life has improved I'm not making myself ill doing things I didn't know were doing that." [P3203]*

> *"…the relief to at that point to speak to a professional who was actually acknowledging that this was happening and you know, it wasn't all in my head. I don't know there, for months and months and months I think it was common there was just no-one apart from my GP, there was just no of recognition or acknowledgement of what people were going through or anything like that. So, it was just such a relief to speak to somebody who was just genuinely interested and very supportive." [P4107]*

In the two health boards where dedicated Long COVID rehabilitation teams existed, AHPs reported that the MDT approach worked well. It was perceived to increase communication, enabling staff to develop both informal learning about Long COVID through peer discussions and also to develop their knowledge and skills in working with the emerging Long COVID clinical population through in-service learning sessions. The Long COVID leads in these areas perceived that the services were valued by people receiving their care and support, which was corroborated by the patient data.

> *"It was my GP that referred me to the psychologist then obviously the two of them were interacting so that the [COVID team] were speaking to the psychologist as well and just basically building a care package for me, so it was tailored to what I was needing physically and mentally, so they were quite good actually talking to each other as well, which was I suppose positive." [P4105]*

For peer review only - http://bmjopen.bmj.com/site/about/guidelines.xhtml

AHPs and Long COVID leads from all health boards reported high levels of staff engagement in learning about Long COVID. In the absence of specific evidence-based guidance on Long COVID rehabilitation, AHPs reported drawing on information from a wide variety of sources. Sources included a sparse literature that had been published on rehabilitation, guidance from professional bodies such as the Chartered Society of Physiotherapy and Royal College of Occupational Therapists, and learning from established approaches for conditions with similar presenting features, such as functional neurological disorder, chronic fatigue syndrome, and fibromyalgia.

AHPs and Long COVID leads reported that several staff developed an individual professional interest in the condition, worked to increase their knowledge, and sought help to learn from peers locally, across health boards in Scotland, and more widely across the United Kingdom.

> *"England has been incredibly helpful in giving and sharing lots and…you know… even within the LOCO-RISE study that's been a really helpful kind of network and relationship to support." [L3203]*

Long COVID leads in health boards where Long COVID rehabilitation services existed reported that these services enabled increased access to care with appropriate referral routes and adaptable and flexible services. One lead described the merit of a blended model of delivery and believed that telephone triage was a useful method of gaining information about patients' needs.

> *"Do you know, I think the use of the increased use of digital services has been fantastic. It's meant that we've been able to reach people when we wouldn't have normally been able to reach them. It has to be a blended model. It has to be that those people that need face-to-face intervention get it at a time when it's right. But digital advances have really made a big difference for us." [L1101]*

Limitations

All participating health boards experienced considerable challenges in delivering Long COVID rehabilitation services. A key challenge was funding, as dedicated funding for Long COVID services had not been allocated by the Scottish Government Long COVID fund at the time these data were collected. The two health boards that had developed dedicated services used existing funding allocations. The short-term nature of this funding in one health board meant that their limited service could not be sustained, and that service ceased during Round 2 of our data collection. This

For peer review only - http://bmjopen.bmj.com/site/about/guidelines.xhtml

limited investment in developing the structure for Long COVID rehabilitation was highlighted by participants as a considerable limiting factor in service delivery, regardless of whether the health board had developed a dedicated service or not, and the *"historical underinvestment in rehab services" [L2101]* was also highlighted by long COVID leads as a significant limiting factor. Where Long COVID dedicated services had not been developed, services reported that they lacked the staffing capacity to meet the perceived additional service demand.

> *I think we're struggling even staffing wise with. We can't staff wards properly. We can't. You know all these things it's. How will we staff a Long COVID service? How will we? How? How will we best provide that service? The NHS way has always been to kind of just absorb it into existing services." [S1202]*

However, where individuals with Long COVID were seen by existing rehabilitation services these services (described henceforth as 'integrated services') were observed to receive considerably fewer referrals for Long COVID rehabilitation. This appeared to be due to the reluctance in these health board areas to promote services (as discussed above). AHPs in integrated services also reported that their referral criteria were often not appropriate for people with Long COVID, resulting in referrals being rejected as inappropriate as PwLC often did not meet pre-existing service criteria for community rehabilitation. Fewer Long COVID referrals entering the services led to reduced confidence among AHPs in their ability to appropriately manage PwLC symptoms, with some admitting they lacked confidence due to Long COVID being a new and largely unknown condition for which they had no training.

> *"I suppose the other challenge from my point of view is actually just setting up a new service from scratch, so you know that's a big job in itself and setting up without the evidence base without kind of any benchmark you know to work with. And so, trying to do that alongside learning about a new disease you know with information coming out constantly." [S4101]*

AHPs in integrated services also reported a lack of knowledge and confidence in working with people presenting with Long COVID due to the broad range of symptomatology.

> *"Hardest thing – so many different symptoms that you can have with Long COVID." [S2204]*

For peer review only - http://bmjopen.bmj.com/site/about/guidelines.xhtml

Finally, less experienced AHPs in integrated services were reported to lack confidence in dealing with Long COVID cases as they saw fewer PwLC. This meant that in some areas increased pressure was placed on the limited capacity of senior staff.

A limitation within both integrated and dedicated rehabilitation services was the lack of medical input to both integrated and dedicated services and the challenges of onward referral to specialist secondary care.

> *"Having the right staff, having medical support in the team, that's been a big gap I feel, and that's still a gap and I think that's really important given the complexity of patients coming through. We're doing a lot of "safety netting" as AHP's at the moment, but I don't think, you know, that that's our job, necessarily. We've had to take on some of that." [S4101]*

DISCUSSION

We explored the perceptions and experiences of community rehabilitation for Long COVID from the perspectives of patients, AHPs delivering community rehabilitation services, and NHS staff leading on the Long COVID response in four Scottish health boards. We were particularly interested in exploring the barriers and facilitators to Long COVID rehabilitation experienced by these three groups across the four different health boards. We found several barriers to accessing community rehabilitation for Long COVID across both dedicated and integrated services, albeit for different reasons, and not only during periods when non-essential services were restricted. We found that the uncertainties of Long COVID, including reluctance of GPs to diagnose, and the constantly evolving evidence-base were barriers to accessing rehabilitation services, and to AHPs confidently providing Long COVID rehabilitation. We also found that lack of or short-term funding, pre-COVID funding of community rehabilitation services, and reluctance to promote existing services to the Long COVID population were key operational barriers to Long COVID rehabilitation. Facilitators included flexible services with clear referral pathways, MDT working, sourcing professional information, and support from within and outwith health board areas. While patients may experience frustration around accessing services, and the evidence base for Long COVID rehabilitation is not firmly established, support and information received from an empathetic AHP was highly valued.

Barriers to accessing healthcare services for Long COVID have been reported in previous research focusing on GPs [11 24] and the views of people with Long COVID [25]. We have highlighted that specific barriers to accessing community rehabilitation for PwLC are principally related to unclear

For peer review only - http://bmjopen.bmj.com/site/about/guidelines.xhtml

referral pathways and demand-capacity issues. Barriers to accessing the physical rehabilitation workforce are not a new phenomenon; Long COVID has arguably brought this previously 'relatively neglected' workforce into the spotlight [26] and highlighted the need for adequate human resourcing of rehabilitation services. This concurs with Baz et al's [11] study of accessing healthcare support for Long COVID in England, which identified staff having to use existing, already stretched, services to support people with Long COVID. Brennan et al [24] reported that GPs (the principal gatekeepers to community rehabilitation services) found referral to community services challenging to negotiate. We identified a complex interplay of factors that impacted on referral from GP to community rehabilitation, including in some health boards a reluctance to promote services due to existing or anticipated capacity issues. Indeed, the dedicated Long COVID service in our study was quickly overwhelmed, demonstrating the real need for Long COVID rehabilitation, which the current epidemiology literature supports [2 27], and highlighting a capacity issue that needs to be urgently addressed.

There is currently no consensus on the optimal model of service delivery for Long COVID, beyond recommendations for it to be multilevel [14 17], multi-professional [14 28-30], person-centred [28], and adequately resourced [14]. Our findings support these recommendations. Services that are flexible to patients' needs and offer a range of delivery modes were valued by patients and staff. The benefit of multidisciplinary team working was a key theme throughout our findings, from both staff and patients' perspectives. However, the MDTs in our study were comprised of various AHPs and in some cases nurses and psychologists; access to medical specialists, as seen in some Long COVID dedicated MDT services or 'one stop' clinics [17] was notably lacking in the four participating health boards and identified as a limitation by participants.

The uncertainties around Long COVID, its diagnosis (particularly with the withdrawal of widespread testing), and optimal management have all been previously reported [31], and are inevitable given the newness of Long COVID as a condition and the as yet emerging evidence-base. It is encouraging that research on Long COVID rehabilitation is ongoing. It is vital to continue to explore Long COVID rehabilitation service delivery models, as well as the effectiveness of specific rehabilitation interventions, acknowledging that there is unlikely to be one that fits all contexts or geographical locations [14] .

Strengths and limitations

This is the first large scale study to explore community rehabilitation services for Long COVID across the Scottish context. We included the perspectives of those receiving and delivering services, as well as those responsible for their oversight. Data collection took place over two rounds, enabling us to

For peer review only - http://bmjopen.bmj.com/site/about/guidelines.xhtml

capture some of the shifting practices inevitable with a new condition. Several researchers were involved at all stages of data analysis and interpretation, including people with lived experience of Long COVID. The study is not without limitations. We selected the four Scottish health boards based on geographical distinction and service delivery variance, as described in our 2019 national survey [13]. However, by the time we commenced data collection, approaches to Long COVID rehabilitation had already evolved meaning that there were fewer distinct service delivery differences between the four health boards. Nonetheless, our selected health board areas enabled us to investigate differences across the two principal modes of service delivery (dedicated and integrated) in Scotland at the time of writing. While we are confident we interviewed all key Long COVID leads in each participating health board and had high levels of AHP participation, patient recruitment was limited by low numbers of patients accessing Long COVID rehabilitation services.

Implications for practice and research

There is likely to be an ongoing need for community rehabilitation for Long COVID, and no 'one-size-fits-all' model of service delivery. The findings presented here can be used to inform the integration of Long COVID into existing services, or to develop new dedicated rehabilitation services. Either way, service providers are required to provide adequate resource, clear referral and management pathways, and facilitate meaningful MDT working. This study represents the first stage in exploring community rehabilitation for Long COVID. We have also explored the perceptions of GPs and adults with Long COVID who have tried to access rehabilitation services (to be reported elsewhere). A realist evaluation of the services in the four health boards reported is ongoing. This evaluation will inform contextualised, evidence-based recommendations for Long COVID rehabilitation. The transferability of key findings from this Scottish -based study should be considered when considering their implications for different clinical and policy contexts.

CONCLUSION

The organisational delivery of Long COVID community rehabilitation is complex and faces several challenges. We have provided a detailed understanding of the barriers and facilitators to Long COVID community rehabilitation from the perspectives of patients, AHPs, and Long COVID leads. The knowledge presented here can be used by those developing and delivering services for people with Long COVID. Community rehabilitation services have much to offer people with Long COVID, but requires adequate planning, publicity, and resource.

For peer review only - http://bmjopen.bmj.com/site/about/guidelines.xhtml

Acknowledgement

The authors would like to thank Alison Despard and Jane Ormerod from long COVID Scotland who provided insight and guidance throughout this study. We are grateful to all our participants who gave their time to take part in interviews and to the local primary investigators from the health boards; Lynne Frew, Lynn Morrison and Gail Thomson-Patel, for their contributions to the LOCO-RISE study. We extend our sincere gratitude to the rehabilitation and administrative staff for supporting recruitment and data collection, and to Valery Burnett for her assistance with data collection.

Author contributions

KC, ED, JM, LA, JP, JO, AD contributed to the study's conception and design. TT, VB, JC, RM, JS undertook data collection. ED, KC, TT, JC, JS, ES, RM undertook analyses and drafted the first version of the manuscript. ED led future iterations of the manuscript. All authors read and commented on the manuscript and approved the final version of it. The corresponding author attests that all authors meet authorship criteria and that nobody meeting the criteria have been omitted. ED and KC have joint responsibility for the research conduct of the study, had access to the data, and controlled the decision to publish.

Ethics approval

Ethical approval was granted from the Wales Research Ethics Committee 6 [21/WA/0118], and each of the four health boards granted R&D management approval.

Funding statement

This work was supported by the Chief Scientist Office Scotland, grant number COV/LTE/20/29.

REFERENCES

1. NICE SIGN RCGP. COVID-19 rapid guideline: managing the longterm effects of COVID-19 2022 [updated 28 February 2023. Available from: https://www.nice.org.uk/guidance/ng188/resources/covid19-rapid-guideline-managing-the-longterm-effects-of-covid19-pdf-510355157422022.
2. Davis HE, McCorkell L, Vogel JM, et al. Long COVID: major findings, mechanisms and recommendations. *Nat Rev Microbiol* 2023;21(3):133-46. doi: 10.1038/s41579-022-00846-2 [published Online First: 20230113]
3. Office for National Statistics (ONS). released 2 February 2023, ONS website, statistical bulletin, Prevalence of ongoing symptoms following coronavirus (COVID-19) infection in the UK 2023 [Available from:

For peer review only - http://bmjopen.bmj.com/site/about/guidelines.xhtml

https://www.ons.gov.uk/peoplepopulationandcommunity/healthandsocialcare/conditionsanddiseases/bulletins/prevalenceofongoingsymptomsfollowingcoronaviruscovid19infectionintheuk/2february2023 accessed 28 Feburary 2023 2023.
4. NHS England. NHS sets out long COVID action plan for thousands of people with persistent symptom 2022 [Available from: https://www.england.nhs.uk/2022/07/nhs-sets-out-long-covid-action-plan-for-thousands-of-people-with-persistent-symptom/ accessed 20 July 2023 2023.
5. Welsh Government. Funding boost to open long COVID services up to people with long term conditions 2023 [Available from: https://www.gov.wales/funding-boost-open-long-covid-services-people-long-term-conditions accessed 20 July 2023 2023.
6. Scottish Government. Long COVID Support Fund 2021 [Available from: https://www.gov.scot/news/long-covid-support-fund/ accessed 20 July 2023 2023.
7. World Health Organization. Clinical management of COVID-19: living guideline, 13 January 2023. Geneva: World Health Organization, 2023.
8. DeMars J, Brown DA, Angelidis I, et al. What is Safe Long COVID Rehabilitation? *J Occup Rehabil* 2023;33(2):227-30. doi: 10.1007/s10926-022-10075-2
9. NHS England. National commissioning guidance for post COVID services 2022 [Available from: https://www.england.nhs.uk/publication/national-commissioning-guidance-for-post-covid-services/ accessed 28 February 2023 2023.
10. Buttery S, Philip KEJ, Williams P, et al. Patient symptoms and experience following COVID-19: results from a UK-wide survey. *BMJ Open Respir Res* 2021;8(1) doi: 10.1136/bmjresp-2021-001075
11. Baz SA, Fang C, Carpentieri JD, et al. 'I don't know what to do or where to go'. Experiences of accessing healthcare support from the perspectives of people living with Long Covid and healthcare professionals: A qualitative study in Bradford, UK. *Health Expect* 2023;26(1):542-54. doi: 10.1111/hex.13687 [published Online First: 20221213]
12. Macpherson K, Cooper K, Harbour J, et al. Experiences of living with long COVID and of accessing healthcare services: a qualitative systematic review. *BMJ Open* 2022;12(1):e050979. doi: 10.1136/bmjopen-2021-050979 [published Online First: 20220111]
13. Duncan E, Cooper K, Cowie J, et al. A national survey of community rehabilitation service provision for people with long Covid in Scotland. *F1000Res* 2020;9:1416. doi: 10.12688/f1000research.27894.2 [published Online First: 20201207]
14. Decary S, De Groote W, Arienti C, et al. Scoping review of rehabilitation care models for post COVID-19 condition. *Bull World Health Organ* 2022;100(11):676-88. doi: 10.2471/BLT.22.288105 [published Online First: 20221003]
15. Flannery T, Brady-Sawant H, Tarrant R, et al. A Mixed-Methods Evaluation of a Virtual Rehabilitation Program for Self-Management in Post-COVID-19 Syndrome (Long COVID). *Int J Environ Res Public Health* 2022;19(19) doi: 10.3390/ijerph191912680 [published Online First: 20221004]
16. Killingback C, Thompson M, Nettleton M, et al. Telerehabilitation for patients who have been hospitalised with covid-19: a qualitative study. *Disabil Rehabil* 2023:1-9. doi: 10.1080/09638288.2022.2159075 [published Online First: 20230111]
17. Parkin A, Davison J, Tarrant R, et al. A Multidisciplinary NHS COVID-19 Service to Manage Post-COVID-19 Syndrome in the Community. *J Prim Care Community Health* 2021;12:21501327211010994. doi: 10.1177/21501327211010994

For peer review only - http://bmjopen.bmj.com/site/about/guidelines.xhtml

18. Lloyd-Evans PHI, Baldwin MM, Daynes E, et al. Early experiences of the Your COVID Recovery((R)) digital programme for individuals with long COVID. *BMJ Open Respir Res* 2022;9(1) doi: 10.1136/bmjresp-2022-001237
19. Willis DG, Sullivan-Bolyai S, Knafl K, et al. Distinguishing Features and Similarities Between Descriptive Phenomenological and Qualitative Description Research. *West J Nurs Res* 2016;38(9):1185-204. doi: 10.1177/0193945916645499 [published Online First: 20160422]
20. Tong A, Sainsbury P, Craig J. Consolidated criteria for reporting qualitative research (COREQ): a 32-item checklist for interviews and focus groups. *Int J Qual Health Care* 2007;19(6):349-57. doi: 10.1093/intqhc/mzm042 [published Online First: 20070914]
21. Hoffmann TC, Glasziou PP, Boutron I, et al. Better reporting of interventions: template for intervention description and replication (TIDieR) checklist and guide. *BMJ* 2014;348:g1687. doi: 10.1136/bmj.g1687 [published Online First: 20140307]
22. Gale NK, Heath G, Cameron E, et al. Using the framework method for the analysis of qualitative data in multi-disciplinary health research. *BMC Med Res Methodol* 2013;13:117. doi: 10.1186/1471-2288-13-117 [published Online First: 20130918]
23. Goldsmith LJ. Using Framework Analysis in Applied Qualitative Research. 2021
24. Brennan A, Broughan J, McCombe G, et al. Enhancing the management of long COVID in general practice: a scoping review. *BJGP Open* 2022;6(3) doi: 10.3399/BJGPO.2021.0178 [published Online First: 20220928]
25. Kingstone T, Taylor AK, O'Donnell CA, et al. Finding the 'right' GP: a qualitative study of the experiences of people with long-COVID. *BJGP Open* 2020;4(5) doi: 10.3399/bjgpopen20X101143 [published Online First: 20201215]
26. Jesus TS, Landry MD, Dussault G, et al. Human resources for health (and rehabilitation): Six Rehab-Workforce Challenges for the century. *Hum Resour Health* 2017;15(1):8. doi: 10.1186/s12960-017-0182-7 [published Online First: 20170123]
27. Gokani SA, Ta NH, Espehana A, et al. The growing burden of long COVID in the United Kingdom: Insights from the UK Coronavirus Infection Survey. *Int Forum Allergy Rhinol* 2022 doi: 10.1002/alr.23103 [published Online First: 20221208]
28. Ceban F, Leber A, Jawad MY, et al. Registered clinical trials investigating treatment of long COVID: a scoping review and recommendations for research. *Infect Dis (Lond)* 2022;54(7):467-77. doi: 10.1080/23744235.2022.2043560 [published Online First: 20220314]
29. Nguyen NN, Hoang VT, Dao TL, et al. Clinical patterns of somatic symptoms in patients suffering from post-acute long COVID: a systematic review. *Eur J Clin Microbiol Infect Dis* 2022;41(4):515-45. doi: 10.1007/s10096-022-04417-4 [published Online First: 20220210]
30. Ostrowska M, Rzepka-Cholasinska A, Pietrzykowski L, et al. Effects of Multidisciplinary Rehabilitation Program in Patients with Long COVID-19: Post-COVID-19 Rehabilitation (PCR SIRIO 8) Study. *J Clin Med* 2023;12(2) doi: 10.3390/jcm12020420 [published Online First: 20230104]
31. O'Hare AM, Vig EK, Iwashyna TJ, et al. Complexity and Challenges of the Clinical Diagnosis and Management of Long COVID. *JAMA Netw Open* 2022;5(11):e2240332. doi: 10.1001/jamanetworkopen.2022.40332 [published Online First: 20221101]

For peer review only - http://bmjopen.bmj.com/site/about/guidelines.xhtml

Consolidated criteria for reporting qualitative studies (COREQ): 32-item checklist

Please indicate in which section each item has been reported in your manuscript. If you do not feel an item applies to your manuscript, please enter N/A.

For further information about the COREQ guidelines, please see Tong *et al.*, 2017: https://doi.org/10.1093/intqhc/mzm042

No.	Item	Description	Section #
**Domain 1: Research team and reflexivity**			
Personal characteristics			
1.	Interviewer/facilitator	Which author/s conducted the interview or focus group?	
2.	Credentials	What were the researcher's credentials? *E.g. PhD, MD*	
3.	Occupation	What was their occupation at the time of the study?	
4.	Gender	Was the researcher male or female?	
5.	Experience and training	What experience or training did the researcher have?	
Relationship with participants			
6.	Relationship established	Was a relationship established prior to study commencement?	
7.	Participant knowledge of the interviewer	What did the participants know about the researcher? *E.g. Personal goals, reasons for doing the research*	
8.	Interviewer characteristics	What characteristics were reported about the interviewer/facilitator? *E.g. Bias, assumptions, reasons and interests in the research topic*	
**Domain 2: Study design**			
Theoretical framework			
9.	Methodological orientation and theory	What methodological orientation was stated to underpin the study? *E.g. grounded theory, discourse analysis, ethnography, phenomenology, content analysis*	
Participant selection			
10.	Sampling	How were participants selected? *E.g. purposive, convenience, consecutive, snowball*	
11.	Method of approach	How were participants approached? *E.g. face-to-face, telephone, mail, email*	
12.	Sample size	How many participants were in the study?	
13.	Non-participation	How many people refused to participate or dropped out? What were the reasons for this?	
Setting			
14.	Setting of data collection	Where was the data collected? *E.g. home, clinic, workplace*	
15.	Presence of non-participants	Was anyone else present besides the participants and researchers?	

For peer review only - http://bmjopen.bmj.com/site/about/guidelines.xhtml

16.	Description of sample	What are the important characteristics of the sample? *E.g. demographic data, date*	
Data collection			
17.	Interview guide	Were questions, prompts, guides provided by the authors? Was it pilot tested?	
18.	Repeat interviews	Were repeat interviews carried out? If yes, how many?	
19.	Audio/visual recording	Did the research use audio or visual recording to collect the data?	
20.	Field notes	Were field notes made during and/or after the interview or focus group?	
21.	Duration	What was the duration of the interviews or focus group?	
22.	Data saturation	Was data saturation discussed?	
23.	Transcripts returned	Were transcripts returned to participants for comment and/or correction?	
**Domain 3: analysis and findings**			
Data analysis			
24.	Number of data coders	How many data coders coded the data?	
25.	Description of the coding tree	Did authors provide a description of the coding tree?	
26.	Derivation of themes	Were themes identified in advance or derived from the data?	
27.	Software	What software, if applicable, was used to manage the data?	
28.	Participant checking	Did participants provide feedback on the findings?	
Reporting			
29.	Quotations presented	Were participant quotations presented to illustrate the themes / findings? Was each quotation identified? *E.g. Participant number*	
30.	Data and findings consistent	Was there consistency between the data presented and the findings?	
31.	Clarity of major themes	Were major themes clearly presented in the findings?	
32.	Clarity of minor themes	Is there a description of diverse cases or discussion of minor themes?	

When submitting your manuscript via the online submission form, please upload the completed checklist as a Figure/supplementary file.

If you would like this checklist to be included alongside your article, we ask that you upload the completed checklist to an online repository and include the guideline type, name of the repository, DOI and license in the *Data availability* section of your manuscript.

Developed from: Allison Tong, Peter Sainsbury, Jonathan Craig, Consolidated criteria for reporting qualitative research (COREQ): a 32-item checklist for interviews and focus groups, International Journal for Quality in Health Care, Volume 19, Issue 6, December 2007, Pages 349–357, https://doi.org/10.1093/intqhc/mzm042

BMJ Open

Understanding Scottish Long COVID community rehabilitation service models from the perspectives of people living with Long COVID and healthcare professionals: a mixed method case study design

Journal:	*BMJ Open*
Manuscript ID	bmjopen-2023-078740.R1
Article Type:	Original research
Date Submitted by the Author:	12-Oct-2023
Complete List of Authors:	Duncan, Edward; University of Stirling, NMAHP-RU<br>Alexander, L; Scottish Centre for Evidence-Based Multi-Professional Practice; Robert Gordon University, School of Health Sciences<br>Cowie, Julie; Glasgow Caledonian University, NMAHP Research Unit<br>Love, Alison; Robert Gordon University<br>Morris, Jacqui H.; University of Dundee, SNHS<br>Moss, Rachel; Healthcare Improvement Scotland<br>Ormerod, Jane; Robert Gordon University<br>Preston, Jenny; Ayrshire Central Hospital, Douglas Grant Rehabilitation Centre<br>Shim, Joanna; Robert Gordon University, School of Health Sciences<br>Stage, Emma; Robert Gordon University, School of Health Sciences<br>Tooman, Tricia; University of Stirling, NMAHP-RU<br>Cooper, K; Robert Gordon University, School of Health Sciences
**Primary Subject Heading**:	Rehabilitation medicine
Secondary Subject Heading:	Evidence based practice
Keywords:	Post-Acute COVID-19 Syndrome, HEALTH SERVICES ADMINISTRATION & MANAGEMENT, REHABILITATION MEDICINE

SCHOLARONE™
Manuscripts

I, the Submitting Author has the right to grant and does grant on behalf of all authors of the Work (as defined in the below author licence), an exclusive licence and/or a non-exclusive licence for contributions from authors who are: i) UK Crown employees; ii) where BMJ has agreed a CC-BY licence shall apply, and/or iii) in accordance with the terms applicable for US Federal Government officers or employees acting as part of their official duties; on a worldwide, perpetual, irrevocable, royalty-free basis to BMJ Publishing Group Ltd ("BMJ") its licensees and where the relevant Journal is co-owned by BMJ to the co-owners of the Journal, to publish the Work in this journal and any other BMJ products and to exploit all rights, as set out in our licence.

The Submitting Author accepts and understands that any supply made under these terms is made by BMJ to the Submitting Author unless you are acting as an employee on behalf of your employer or a postgraduate student of an affiliated institution which is paying any applicable article publishing charge ("APC") for Open Access articles. Where the Submitting Author wishes to make the Work available on an Open Access basis (and intends to pay the relevant APC), the terms of reuse of such Open Access shall be governed by a Creative Commons licence – details of these licences and which Creative Commons licence will apply to this Work are set out in our licence referred to above.

Other than as permitted in any relevant BMJ Author's Self Archiving Policies, I confirm this Work has not been accepted for publication elsewhere, is not being considered for publication elsewhere and does not duplicate material already published. I confirm all authors consent to publication of this Work and authorise the granting of this licence.

For peer review only

TITLE: Understanding Scottish Long COVID community rehabilitation service models from the perspectives of people living with Long COVID and healthcare professionals: a mixed method case study design

ABSTRACT

Objectives: To explore the perceptions and experiences of barriers and facilitators to accessing Long COVID community rehabilitation.

Design: We used a qualitative descriptive design over two rounds of data collection with three participant groups: i) people with experience of rehabilitation for Long COVID (PwLC); ii) NHS staff delivering and/or managing community rehabilitation services (allied health professionals (AHPs)), and iii) NHS staff involved in strategic planning around Long COVID in their health board (Long COVID leads).

Setting: Four NHS Scotland territorial health boards.

Participants: 51 interviews: eight Long COVID leads (11 interviews); 15 AHPs (25 interviews) and 15 PwLC (15 interviews).

Results: Three key themes were identified: i) Accessing care for PwLC, ii) Understanding Long COVID and its management; and iii) Strengths and limitations of existing Long COVID rehabilitation services.

Conclusions: Organisational delivery of Long COVID community rehabilitation is complex and presents multiple challenges. In addition, access to Long COVID community rehabilitation can be challenging. When accessed, these services are valued by PwLC but require adequate planning, publicity, and resource. The findings presented here can be used by those developing and delivering services for people with Long COVID.

Strengths and limitations of this study

- This study was designed to explore the development and implementation of long covid rehabilitation services in four contrasting regions of Scotland from key stakeholder perspectives
- Case study design enabled an analysis of development of differing service models of delivery over the same time period
- The rapidly evolving nature of Long COVID and its management resulted in fewer distinct differences between the four health boards than originally anticipated
- Other relevant perspectives, such as General Practice/family medicine are not included in this study but will be presented in subsequent publications.

For peer review only - http://bmjopen.bmj.com/site/about/guidelines.xhtml

INTRODUCTION

Background

Long COVID, a condition defined as ongoing COVID-19 symptoms that continue beyond twelve weeks following an initial acute COVID-19 infection, was first described in Spring 2020 [1]. It is estimated that at least 65 million people globally have Long Covid [2]. By January 2023, over 2 million people were estimated to have Long COVID in the United Kingdom (UK), including over 175,000 living in Scotland [3]. Long COVID has broad multiple system presentation and can have profound physical, emotional, social, and financial consequences. Moreover, debilitating symptoms of Long COVID often persist for a year or more [2].

NHS funding to support the diagnosis, treatment, and rehabilitation for Long COVID varies across the UK. Since the autumn of 2020, NHS England have allocated £94 million (£1.66 per capita, based on the 2021 UK Office of National Statistics Census data England's population was 56,489,800) to be invested in specialist Long COVID clinics in England to complement existing primary, community, and rehabilitation care services [4]. The Welsh Government has allocated £18.3 million since 2020 (£5.88 per capita based on a population of 3,107,500 in 2021) to support the delivery of Long COVID diagnosis, treatment, and rehabilitation within existing services [5]. In September 2021, the Scottish Government announced £10 million (£1.82 per capita based on a population of 5,479,900 in 2021) over 3 years to support Scottish NHS health boards' response to Long COVID [6]. At the end of May 2022, plans for the first £3 million of that funding were announced.

The National Institute for Health and Care Excellence (NICE) [1] and The World Health Organisation [7] recommend people with Long COVID, following an initial medical assessment to exclude underlying conditions, have access to rehabilitation to aid their recovery. As with other long-term conditions, community rehabilitation for people with Long COVID should be personalised, multidisciplinary, and comprehensive in order to maximise function, quality of life, and participation in society [8 9].

However, the optimal approach to deliver Long COVID community rehabilitation is currently unknown. NICE clinical guidance suggests broad rehabilitation approaches, which can be delivered in isolation or in combination, such as self-management and multidisciplinary rehabilitation [1]. The effectiveness of these approaches is not yet clear and there are further uncertainties about the most appropriate means of organising service delivery, particularly that of multidisciplinary rehabilitation. There are well-documented barriers to accessing healthcare services for PwLC [10-12]; however previous research has not specifically considered rehabilitation services. Our 2019 national survey [13] of Scotland's 14 territorial health boards found that Long COVID community

For peer review only - http://bmjopen.bmj.com/site/about/guidelines.xhtml

rehabilitation was universally available across Scotland. At the time of the survey, one health board was delivering Long COVID community rehabilitation as a dedicated service, with the remainder integrating rehabilitation within existing services. And yet, there was substantial variation across health boards in the mode of service delivery, given that the means of optimally delivering community rehabilitation to this population was unknown.

To date rehabilitation services for Long COVID have varied internationally with respect to delivery and the healthcare professionals involved [14], including virtual rehabilitation focusing on self-management and education [15], group-based pulmonary rehabilitation [16], tiered multidisciplinary pathways [17], and phased approaches to managing symptoms [18]. Our research group are investigating models of service delivery for Long COVID rehabilitation in a study called LOCO-RISE, which will provide recommendations for the optimal provision of community rehabilitation for PwLC. As part of our larger study, we explored the experiences and perceptions of people receiving rehabilitation for Long COVID and NHS staff (operational and strategic) involved in delivering or managing Long COVID community rehabilitation. The aim of this phase of LOCO-RISE was to explore the perceptions and experiences of barriers and facilitators to implementing Long COVID community rehabilitation, from the perspectives of all key stakeholders in relation to delivering and receiving Long COVID rehabilitation, as services evolved over a 12-month period.

METHODS

Study Design

This paper reports on the first two rounds of data collection of a longitudinal qualitative descriptive [19] study based on 51 interviews with three key participant groups, each of which provided distinct perspectives; i) people with experience of rehabilitation for Long COVID (PwLC), who provided lived experiences of receiving Long Covid rehabilitation; ii) NHS staff who provided perspectives of delivering and/or managing community rehabilitation services for PwLC (allied health professionals (AHPs)), and iii) NHS staff involved in strategic planning around Long COVID in their health board (Long COVID leads) who provided broader organisational perspectives. The study is reported in accordance with the Consolidated criteria for reporting qualitative research [COREQ] [Supplemental File 1])[20]. Qualitative description was selected over other qualitative designs as it enabled the exploration of a range of shared experiences to generate a rich description [19] of barriers and facilitators to the implementation of Long COVID rehabilitation.

For peer review only - http://bmjopen.bmj.com/site/about/guidelines.xhtml

Setting

The study was conducted in four Scottish health boards (HB1-HB4). The boards were selected for their geographical and demographic spread as well as variation in Long COVID rehabilitation service models, summarised in Table 1. As shown in Table 1, all boards adopted some form of multi-disciplinary team (MDT) approach using a blended mode of delivery (face-to-face, telephone, Near Me). Staffing varied; occupational therapy and physiotherapy were most consistent across services, with some including additional professions such as dietetics, speech and language therapy, and psychology. Some staff were co-located, while others worked as part of a team, but geographically remote. Notably, none of the services included medical staff.

For peer review only

Table 1: Description of participating health boards

Health Board	Health Board 1	Health Board 2	Health Board 3	Health Board 4
**Setting**	Largely rural	Large urban/rural area	Largely urban	Large urban/rural area
**Round 1 Long COVID service delivery model**	Integrated service: community rapid response rehabilitation	Integrated service: community rehabilitation	Integrated service: community rehabilitation	Dedicated long COVID service
	**MDT:** Physiotherapy, Occupational Therapy, Nursing, Community Psychiatric Nurse (CPN), Pharmacy, Advanced Nurse Practitioner (ANP), and Speech and Language Therapy, with links to GP and Dietetics	**MDT:** Physiotherapy, Occupational Therapy, Dietetics, and Speech and Language Therapy.	**MDT:** Physiotherapy, Occupational Therapy, Psychology, Speech and Language Therapy, and Rehabilitation Assistant Practitioner, Physiotherapy, Occupational Therapy, Psychology, Dietetics, and Speech and Language Therapy	**MDT:** Physiotherapy, Occupational Therapy, Psychology, Dietetics, and Speech and Language Therapy
	Blended delivery	Blended delivery with centralised point of referral	Blended delivery	Blended delivery
**Round 2 Long COVID service delivery model**	As Round 1	As Round 1	Integrated service moving towards a soft launch of dedicated long COVID Service<br><br>Blended delivery with single access point	Dedicated Long COVID Service via a 3-tiered triage system<br><br>Service temporarily halted due to large volume of referrals and subsequent decline in staffing and funding

Key: MDT=Multidisciplinary

For peer review only - http://bmjopen.bmj.com/site/about/guidelines.xhtml

Recruitment and Data Collection

Two rounds of data collection took place: Round 1 (November 2021 – April 2022) and Round 2 (June – July 2022). In each round, three participant groups took part: PwLC, AHPs, and Long COVID leads. Long COVID leads were purposively sampled by email invitation or word of mouth, for their role in leading on Long COVID within their health board, and included AHP directors, medical directors, and clinical service leads. Long COVID leads who only managed in-patient services were ineligible, since this study was exploring community rehabilitation. AHPs, most often occupational therapists and physiotherapists, were involved in providing Long COVID rehabilitation. Most AHPs acted in a team member role, though a small number were also clinical service leads. AHPs involved in the treatment of PwLC in the study health board areas were purposively recruited by email invitation. The invitation was sent by senior managers on behalf of the study team and explained the reasons for the research. Student AHPs were ineligible, as were AHPs who only provided in-patient Long COVID rehabilitation. Finally, a convenience sample of PwLC were recruited by their AHP. PwLC were provided with a study pack in printed or electronic format that explained the purpose of the study and volunteers opted into the study by contacting the research team by email, telephone, or leaving their contact details after completing a survey of health-related outcomes as part of the larger study. Patients were ineligible if they were aged under 18, required specialist (in-patient) management of Long COVID complications, had another life-limiting illness with reduced life-expectancy (e.g., disseminated malignancy) or were unable or unwilling to provide informed consent. Purposive sampling criteria were informed by prior knowledge of the emerging Long COVID services in each Health Board. We aimed to recruit 12 AHPs, 4 -8 Long COVID leads and up to 32 patients for each round of data collection.

Interview topic guides [Supplemental file 2] were developed by the research team that included people with lived experience of Long COVID. Topic guides were further refined for Round 2 to take account of the temporal nature of the questions being asked. The topic guides combined a theory-based approach using the TIDieR intervention description categories [21] to enable a comprehensive description of each rehabilitation service model, which can be conceived of as an organisational level intervention. Questions to long COVID leads were based on constructs from the Consolidated Framework for Implementation Research [22] to ascertain the factors that were associated with the intervention’s implementation and a series of questions exploring facilitators and barriers to delivering and receiving community Long COVID rehabilitation. The interview topic guides were pilot tested with people who had lived experienced of Long Covid and with two AHPs who were not involved in the study prior to implementation. Web-based [Microsoft Teams] or telephone interviews were conducted by five experienced healthcare researchers with master’s or doctoral

For peer review only - http://bmjopen.bmj.com/site/about/guidelines.xhtml

level qualifications and experience of qualitative research [VB, JC, RM, JS, TT]. All interviewers were female and had experience of qualitative NHS data collection and analysis. Two of the researchers were AHPs and had prior awareness of the challenges of delivering rehabilitation. All interviewers were provided with training and supervision from KC and ED and had no prior relationship with the services or participants. Informed consent was obtained from all participants and recorded verbally prior to each interview. Interviews lasted between 22 and 86 minutes (average 52 minutes). Only the participant and interviewer were present during each interview. Interviews were audio recorded and transcribed intelligently by an external transcription service, where redundant words and/or sounds were removed. Field notes were taken during some interviews. These notes added to team discussions to support understanding of the data as it was collected but did not form part of the official data analysis. Transcripts were not returned to participants for comment or correction.

Data analysis

Data were analysed using the Framework method, which is commonly used in applied health research and recognised as appropriate for multidisciplinary research teams [23]. As a form of thematic analysis, the framework approach was congruent with the qualitative descriptive design being employed [19].

For each participant group, at least two researchers from the team familiarised themselves with at least 20% of transcripts, making analytical notes directly on the transcripts. The researcher pairs were from different disciplinary backgrounds (occupational therapy, physiotherapy, psychology, applied health research), thereby ensuring interpretation from a range of perspectives. Following discussion between all team members involved in familiarisation, a framework for analysis and interpretation was constructed [24]. The use of multiple researchers at this stage aimed to mitigate for bias in interpretation that may have occurred due to the disciplinary backgrounds of some team members. Although line-by-line coding is common, it is also possible to develop a framework without engaging in explicit coding [24]; we adopted the latter approach. The 'analytical framework' [23] was applied across all interview transcripts using highlighting and the comments function in Microsoft Word, with regular review and discussion within the team. A charting matrix was created in Microsoft Excel that involved summarising broad categories of data from the transcripts, then interpreting the charted data by exploring within and between-cases to derive concepts and themes. These were then grouped into overarching themes. All researchers were involved in interpreting the data during regular team discussions. We did not assess data saturation, due to the applied Framework approach being used, but considered 'information power' [25] during the analysis process. Participants did not directly provide feedback on the findings. However, the findings were

For peer review only - http://bmjopen.bmj.com/site/about/guidelines.xhtml

presented in a webinar attended by Long Covid Leads, AHPs and PwLC, several of whom had participated in the study. The webinar attendees endorsed the study findings and reflected that they resonated with their personal experiences.

Patient and Public Involvement

Two people with lived experience of Long COVID were core members of the research team. They co-developed study materials and interview topic guides and contributed to analysis and interpretation of study findings.

RESULTS

Participant characteristics

We recruited eight Long COVID leads, who took part in 11 interviews over the two rounds of data collection: 15 AHPs (25 interviews) and 15 PwLC (15 interviews). We did not meet our recruitment target for PwLC despite a high conversion rate (invitation to take part in the study resulting in consent and participation). The low recruitment rate occurred as a result of apparent barriers to PwLC being referred to the community rehabilitation services, a phenomenon that was subsequently explored in a related study (to be reported elsewhere). See Table 2 for participant details. No participants who consented withdrew from the study.

For peer review only

Table 2 Participant demographics

	Long COVID leads (L)	Staff delivering service/AHPs (S)		Patients (P)
	**N=6**<br>**Rounds 1 & 2 N=3**<br>**Round 1 N=2**<br>**Round 2 N=1**	**N=15**<br>**Rounds 1 & 2 N=9**<br>**Round 1 N=2**<br>**Round 2 N=4**		**N=15**
**Age:**				
Working age [%]	8 [100]	15 [100]		13 [87]
		AfC Band	**N=**	
		6	7	
		7	4	
		8a	4	
**Sex:**				
Female [%]	6 [75]	15 [100]		12 [80]
Male [%]	2 [25]	0		3 [20]
**Employment status:**				
Working full-time [%]	8 [100]	15 [100]		6 [40]
Working part-time [%]	0	0		1 [7]
Phased return to work [%]	0	0		2 [13]
Retired [%]	0	0		2 [13]
Unable to work because of illness [%]	0	0		3 [20]
Not provided [%]	0	0		1 [7]
**Time from first COVID diagnosis**	NA	NA		12 – 28 months

Key: AHPs = Allied Health Professionals; AfC = Agenda for Change

For peer review only

Findings

Framework analysis resulted in three key themes being identified: i) Accessing care for PwLC, ii) Understanding Long COVID and its management; and iii) Strengths and limitations of existing long COVID rehabilitation services. Table 3 illustrates the three themes and the categories of data that contributed to each.

Table 3: Key themes and categories of data

Key Themes	Sub themes
**Accessing care for people with Long COVID**	PwLC accessing General Practitioner (GP) services [L; P]<br>GP referring PwLC to community rehabilitation [L; P; S]<br>Awareness of services by GPs & PwLC [S]<br>Signposting [S]<br>Waiting lists & waiting times [P]
**Understanding Long COVID and its management**	Long COVID prevalence [L]<br>Long COVID unknowns [L; P]<br>Reluctance to diagnose [P]<br>Tests/investigations [P]<br>Being believed [P]<br>Evolving evidence-base [L; S]<br>Education of PwLC and community rehabilitation staff [S]<br>Information sharing [S]<br>Learning from other conditions [S]<br>Confidence of community rehabilitation staff [S]
**Long COVID services**	Current services strengths & limitations [L; P; S]<br>Resourcing services [L; P; S]<br>Need for Long COVID services [L; P; S]<br>Navigating politics [L]

Key: L = Long Covid leads; P = PwLC; S = AHPs

Participant data was coded and reported as follows: (1) Role of participant: L = Long Covid leads; P = PwLC; S = AHPs; (2) Health Board (HB) number: HBs 1-4; (3) Round of data collection: 1 or 2; and (4) Participant number (e.g. the participant identifier for the first quote: P-3-2-08 is a PwLC from HB3, data was collected in Round 2 and was the 8th PwLC interviewed).

For peer review only

I: Accessing care for People with Long COVID

Barriers to accessing Long COVID rehabilitation were identified by all participant groups. In Round 1, patients commonly attributed this to limited availability of face-to-face GP appointments due to COVID-19 restrictions.

> *"You don't get face-to-face with the GP. So again, when you get a GP, it's always a different GP you're talking to and you try your best, but you get locum, and they've all been fantastic. I'm not, again, I wouldn't criticise them, but it's exhausting telling the same story every single time." [P3208]*

Long COVID leads and AHPs observed people's reluctance to seek help from services widely acknowledged as being under pressure. By Round 2, they felt that the perception of GPs being closed, and services overwhelmed, needed to be questioned, and called for greater public awareness of Long COVID rehabilitation services and how to access them.

> *"[We] need to get past the public's perception that GPs are shut so they return to their GP and get a referral to their service if required." [L3102]*

All participant groups were aware of the dedicated Long COVID service in HB4. However, due to the publicity the service had received and the pro-active development of a clear GP referral pathway, demand for this service quickly exceeded capacity. Staff (AHPs and Long COVID leads) expressed concern that promoting a Long COVID service in their own board may result in the same situation. Their concerns were compounded by the widely acknowledged pressure in the healthcare system due to a range of factors including staff redeployment, absence, and reopening of non-essential rehabilitation services that had been closed during previous phases of the pandemic.

> *"They* [HB4 Long COVID service] *were basically inundated. So, I had to wait, about four months or so, something like that for a slot. But it was quicker than I thought, but they were just getting overwhelmed with the workload." [P4101*]

> *"Yeah. So, it's [integrated service] not a widely publicised thing, because I don't know if we could cope as a service with the numbers." [S1102]*

For peer review only - http://bmjopen.bmj.com/site/about/guidelines.xhtml

For peer review only

Patients and staff reported both frustration and understanding around long waiting times for Long COVID rehabilitation:

> *"The referral side of it they probably done all the right things, but the timescales were just probably horrific but understandable. I know that in hindsight now so, but it didn't take away the frustrations of it." [P3208]*

> *"Difficult due to capacity. Everywhere so busy and up against it. The pressures on us are really extreme." [L3101]*

The dedicated service in HB4 was accessed by self-referral and delivered by AHP staff. Self-referral was viewed positively by patients; however, staff reported that the lack of information on self-referrals made appropriate triage of patients challenging. Health boards without self-referral and/or a dedicated Long COVID service also expressed concern that people with Long COVID would not be prioritised for rehabilitation within usual community rehabilitation services.

> *"I don't have self-referral at the moment. And the referrals coming through the GP or through something, some of them are coming through. But I don't know where all the others are, so, I'm not blocking access, but they are getting stuck in the system, in my system, in that if I don't have enough information on the referral, they're just going on to the normal routine waiting list." [S2207]*

HB4 reported the lack of medical staff as a limitation to their dedicated service and, in keeping with other health boards, reported that the lack of a clear Long COVID pathway was a limitation, particularly as people often presented with complex issues that were unsuitable for immediate rehabilitation.

> *"But it's those that have the more complex needs that I wouldn't know where to send them to." [S2103]*

II: Understanding Long COVID and its management

Patients commented on the burdensome nature of managing Long COVID and there being a reluctance among GPs to provide a diagnosis. They noted concerns about documenting Long COVID in healthcare records and a need to rule out other underlying conditions.

For peer review only - http://bmjopen.bmj.com/site/about/guidelines.xhtml

"It's quite difficult and just feel like, you know, you have a list as long as your arm when you actually speak to the GP because some things have changed and you know they're very sympathetic, but very clear and honest that well, we don't really know, you know? We just don't know. So, we'll give you this pill to try and treat this symptom at the moment and, that's quite hard.' [P4101]

The lack of diagnosis was also observed by AHPs and Long COVID leads, who shared a discomfort associated with labelling patients.

"What are we shaming people for having a condition? What's that all about? It's very bizarre. You get them help. I don't understand. It's like invisible illnesses." [L1202]

"It was really difficult actually to know whether to adopt the term Long COVID 'cause it's not a medical term, but we've decided in the end it's probably got wider recognition, now, that's what the patients are using. But having to be very careful, actually even when you're writing letters to GPs, you know if they didn't have confirmed COVID in the beginning, it's a presumed COVID illness, you know symptoms consistent with Long COVID, you know. It's a lot of kind of working around the houses I feel." [S4101]

Most patients expressed a need to be heard and believed by healthcare professionals. They also felt that there was powerful value in hearing that other people with Long COVID experienced similar symptoms, which provided a helpful process in validating patients' unmet needs.

"It felt to me like it was a bunch of very random symptoms. It didn't seem connected, and I think probably one of the best things in that first conversation was just hearing that the symptoms I have were very common and they are all related." [P4102]

"I get the impression that a lot of my symptoms are replicated a lot across, across other people so and I'm getting a lot of assurance on what people's progress like recovery-wise again its always pinned by keep your expectations to a minimum, like it's no [not] *going to be the miracle cure." [P3208]*

Both AHPs and Long COVID leads spoke of the challenges of understanding Long COVID and its constantly evolving evidence base, as well as the lack of data on prevalence. AHPs also reported a

For peer review only - http://bmjopen.bmj.com/site/about/guidelines.xhtml

lack of evidence to support best practice for managing Long COVID, which could result in anxiety around patient management.

> *"So, what I was concerned about was what if I give the wrong advice or the wrong sort of exercise prescription, and that I actually cause harm to him by something that I've done. And I watched some of the podcasts to sort of understand what we should and shouldn't be doing, and that is when I started to realize that there is a really strong link with Long COVID and the chronic fatigue syndrome and ME population who have been saying for years and years that, actually, sometimes, graded exercise therapy is really harmful, and you absolutely should not prescribe it." [S2104]*

To increase understanding of Long COVID, AHPs sought out peer support, online resources, and knowledge from other conditions to determine how to support people with Long COVID.

> *"There's certainly discussions around what is the best way to approach management of Long COVID and there's been a few things we can do, though, because nationally there's a huge focus on physiotherapy management of Long COVID you know, the CSP [Chartered Society of Physiotherapists], I've got lots of stuff out there, but we've also got the clinicians and the COVID rehab team there that although they're not seeing patients and more than happy to signpost people to resources and help with a bit of professional advice. I would say from a Community hub, clinician point of view, confidence hasn't been the main issue. They do seek professional advice on how to manage them." [S3203]*

III: Strengths and limitations of existing Long COVID services

A characteristic of all participating health boards during the study period was the dynamically evolving nature of the services provided. This was partly due to external factors relating to COVID prevalence, the restrictions this placed on service delivery, and financial planning decisions. Two health board areas changed their organisational structures affecting services. One health board (HB4) commenced a limited dedicated Long COVID rehabilitation service, delivered by an occupational therapist and a physiotherapist. The service developed clear pathways for referral that gained considerable publicity, as a result of which, the service was unable to meet demand. This was compounded by staff leaving their post. A considerable waiting list developed. The service ran for 18 months after which there was no further funding to continue its delivery, the service ceased and individuals on the waiting list were distributed to local community rehabilitation teams.

For peer review only - http://bmjopen.bmj.com/site/about/guidelines.xhtml

Long Covid service strengths

By Round 2 of data collection, AHPs in all health boards were able to see PwLC face-to-face, conduct home visits, have greater integration into the community, and begin working towards return-to-work packages with appropriate Long COVID patients. All these service features were perceived by clinicians to increase the flexibility of service delivery. Patients had experienced a range of modes of service delivery, largely a blended model of face-to-face individual, group, or online interventions. However, irrespective of the delivery model, most patients had a positive experience of Long COVID rehabilitation, especially the self-management information they received. PwLC reported that validation of their experience was particularly significant.

> *"I'm certainly more educated, already daily life has improved I'm not making myself ill doing things I didn't know were doing that." [P3203]*

> *"…the relief to at that point to speak to a professional who was actually acknowledging that this was happening and you know, it wasn't all in my head. I don't know there, for months and months and months I think it was common there was just no-one apart from my GP, there was just no of recognition or acknowledgement of what people were going through or anything like that. So, it was just such a relief to speak to somebody who was just genuinely interested and very supportive." [P4107]*

In the two health boards where dedicated Long COVID rehabilitation teams existed, AHPs reported that the MDT approach worked well. It was perceived to increase communication, enabling staff to develop both informal learning about Long COVID through peer discussions and also to develop their knowledge and skills in working with the emerging Long COVID clinical population through in-service learning sessions. The Long COVID leads in these areas perceived that the services were valued by people receiving their care and support, which was corroborated by the patient data.

> *"It was my GP that referred me to the psychologist then obviously the two of them were interacting so that the [COVID team] were speaking to the psychologist as well and just basically building a care package for me, so it was tailored to what I was needing physically and mentally, so they were quite good actually talking to each other as well, which was I suppose positive." [P4105]*

For peer review only - http://bmjopen.bmj.com/site/about/guidelines.xhtml

AHPs and Long COVID leads from all health boards reported high levels of staff engagement in learning about Long COVID. In the absence of specific evidence-based guidance on Long COVID rehabilitation, AHPs reported drawing on information from a wide variety of sources. Sources included a sparse literature that had been published on rehabilitation, guidance from professional bodies such as the Chartered Society of Physiotherapy and Royal College of Occupational Therapists, and learning from established approaches for conditions with similar presenting features, such as functional neurological disorder, chronic fatigue syndrome, and fibromyalgia.

AHPs and Long COVID leads reported that several staff developed an individual professional interest in the condition, worked to increase their knowledge, and sought help to learn from peers locally, across health boards in Scotland, and more widely across the United Kingdom.

> *"England has been incredibly helpful in giving and sharing lots and…you know… even within the LOCO-RISE study that's been a really helpful kind of network and relationship to support." [L3203]*

Long COVID leads in health boards where Long COVID rehabilitation services existed reported that these services enabled increased access to care with appropriate referral routes and adaptable and flexible services. One lead described the merit of a blended model of delivery and believed that telephone triage was a useful method of gaining information about patients' needs.

> *"Do you know, I think the use of the increased use of digital services has been fantastic. It's meant that we've been able to reach people when we wouldn't have normally been able to reach them. It has to be a blended model. It has to be that those people that need face-to-face intervention get it at a time when it's right. But digital advances have really made a big difference for us." [L1101]*

Long Covid service limitations

All participating health boards experienced considerable challenges in delivering Long COVID rehabilitation services. A key challenge was funding, as dedicated funding for Long COVID services had not been allocated by the Scottish Government Long COVID fund at the time these data were collected. The two health boards that had developed dedicated services used existing funding allocations. The short-term nature of this funding in one health board meant that their limited service could not be sustained, and that service ceased during Round 2 of our data collection. This

For peer review only - http://bmjopen.bmj.com/site/about/guidelines.xhtml

limited investment in developing the structure for Long COVID rehabilitation was highlighted by participants as a considerable limiting factor in service delivery, regardless of whether the health board had developed a dedicated service or not, and the *"historical underinvestment in rehab services" [L2101]* was also highlighted by long COVID leads as a significant limiting factor. Where Long COVID dedicated services had not been developed, services reported that they lacked the staffing capacity to meet the perceived additional service demand.

> *I think we're struggling even staffing wise with. We can't staff wards properly. We can't. You know all these things it's. How will we staff a Long COVID service? How will we? How? How will we best provide that service? The NHS way has always been to kind of just absorb it into existing services." [S1202]*

However, where individuals with Long COVID were seen by existing rehabilitation services these services (described henceforth as 'integrated services') were observed to receive considerably fewer referrals for Long COVID rehabilitation. This appeared to be due to the reluctance in these health board areas to promote services (as discussed above). AHPs in integrated services also reported that their referral criteria were often not appropriate for people with Long COVID, resulting in referrals being rejected as inappropriate as PwLC often did not meet pre-existing service criteria for community rehabilitation. Fewer Long COVID referrals entering the services led to reduced confidence among AHPs in their ability to appropriately manage PwLC symptoms, with some admitting they lacked confidence due to Long COVID being a new and largely unknown condition for which they had no training.

> *"I suppose the other challenge from my point of view is actually just setting up a new service from scratch, so you know that's a big job in itself and setting up without the evidence base without kind of any benchmark you know to work with. And so, trying to do that alongside learning about a new disease you know with information coming out constantly." [S4101]*

AHPs in integrated services also reported a lack of knowledge and confidence in working with people presenting with Long COVID due to the broad range of symptomatology.

> *"Hardest thing – so many different symptoms that you can have with Long COVID." [S2204]*

For peer review only - http://bmjopen.bmj.com/site/about/guidelines.xhtml

Finally, less experienced AHPs in integrated services were reported to lack confidence in dealing with Long COVID cases as they saw fewer PwLC. This meant that in some areas increased pressure was placed on the limited capacity of senior staff.

A limitation within both integrated and dedicated rehabilitation services was the lack of medical input to both integrated and dedicated services and the challenges of onward referral to specialist secondary care.

> *"Having the right staff, having medical support in the team, that's been a big gap I feel, and that's still a gap and I think that's really important given the complexity of patients coming through. We're doing a lot of "safety netting" as AHP's at the moment, but I don't think, you know, that that's our job, necessarily. We've had to take on some of that." [S4101]*

DISCUSSION

We explored the perceptions and experiences of community rehabilitation for Long COVID from the perspectives of patients, AHPs delivering community rehabilitation services, and NHS staff leading on the Long COVID response in four Scottish health boards. We were particularly interested in exploring the barriers and facilitators to Long COVID rehabilitation experienced by these three groups across the four different health boards. We found several barriers to accessing community rehabilitation for Long COVID across both dedicated and integrated services, albeit for different reasons, and not only during periods when non-essential services were restricted. We found that the uncertainties of Long COVID, including perceived reluctance of GPs to diagnose, and the constantly evolving evidence-base were barriers to accessing rehabilitation services, and to AHPs confidently providing Long COVID rehabilitation. We also found that lack of or short-term funding, limited pre-COVID funding of community rehabilitation services, and reluctance to promote existing services to the Long COVID population were key operational barriers to Long COVID rehabilitation service provision. Facilitators included flexible services with clear referral pathways, MDT working, sourcing professional information, and support from within and outwith health board areas. While PwLC may experience frustration around accessing services and the evidence base for Long COVID rehabilitation is not firmly established, support and information received from an empathetic AHP was highly valued.

Barriers to accessing healthcare services for Long COVID have been reported in previous research focusing on GPs [11 26] and the views of people with Long COVID [27]. We have highlighted that

For peer review only - http://bmjopen.bmj.com/site/about/guidelines.xhtml

For peer review only

specific barriers to accessing community rehabilitation for PwLC are principally related to unclear referral pathways and demand-capacity issues. Barriers to accessing the physical rehabilitation workforce are not a new phenomenon; Long COVID has arguably brought this previously 'relatively neglected' workforce into the spotlight [28] and highlighted the need for adequate human resourcing of rehabilitation services. This concurs with Baz et al's [11] study of accessing healthcare support for Long COVID in England, which identified staff having to use existing, already stretched, services to support people with Long COVID. Brennan et al [26] reported that GPs (the principal gatekeepers to community rehabilitation services) found referral to community services challenging to negotiate. We identified a complex interplay of factors that impacted on referral from GP to community rehabilitation, including in some health boards a reluctance to promote services due to existing or anticipated capacity issues. Indeed, the dedicated Long COVID service in our study was quickly overwhelmed, demonstrating the real need for Long COVID rehabilitation, which the current epidemiology literature supports [2 29], and highlighting a capacity issue that needs to be urgently addressed.

There is currently no consensus on the optimal model of service delivery for Long COVID, beyond recommendations for it to be multilevel [14 17], multi-professional [14 30-32], person-centred [30], and adequately resourced [14]. Our findings support these recommendations. Services that are flexible to patients' needs and offer a range of delivery modes were valued by patients and staff. The benefit of multidisciplinary team working was a key theme throughout our findings, from both staff and patients' perspectives. However, the MDTs in our study were comprised of various AHPs and in some cases nurses and psychologists; access to medical specialists, as seen in some Long COVID dedicated MDT services or 'one stop' clinics [17] was notably lacking in the four participating health boards and identified as a limitation by participants.

The uncertainties around Long COVID, its diagnosis (particularly with the withdrawal of widespread testing), and optimal management have all been previously reported [33], and are inevitable given the newness of Long COVID as a condition and the as yet emerging evidence-base. It is encouraging that research on Long COVID rehabilitation is ongoing. It is vital to continue to explore Long COVID rehabilitation service delivery models, as well as the effectiveness of specific rehabilitation interventions, acknowledging that there is unlikely to be one that fits all contexts or geographical locations [14] .

Strengths and limitations

This is the first large scale study to explore community rehabilitation services for Long COVID across the Scottish context. We included the perspectives of those receiving and delivering services, as well

For peer review only - http://bmjopen.bmj.com/site/about/guidelines.xhtml

as those responsible for their oversight. Data collection took place over two rounds, enabling us to capture some of the shifting practices inevitable with a new condition. Several researchers were involved at all stages of data analysis and interpretation, including people with lived experience of Long COVID. Validity of the study findings were therefore enhanced by data (two rounds and three participant groups) and investigator triangulation (multiple researchers involved in analytical decisions) [34 35]. The study is not without limitations. We selected the four Scottish health boards based on geographical distinction and service delivery variance, as described in our 2019 national survey [13]. However, by the time we commenced data collection, approaches to Long COVID rehabilitation had already evolved meaning that there were fewer distinct service delivery differences between the four health boards. Nonetheless, our selected health board areas enabled us to investigate differences across the two principal modes of service delivery (dedicated and integrated) in Scotland at the time of writing. While we are confident we interviewed all key Long COVID leads in each participating health board and had high levels of AHP participation, thereby achieving adequate information power [25] or these two groups patient recruitment was limited by low numbers of patients accessing Long COVID rehabilitation services. This study investigated the perspectives of three key stakeholder groups: PwLC, staff delivering services and Long COVID service leads involved in wider strategic and organisational issues. Other relevant stakeholder's perspectives, such as General Practitioners/family doctors were not included in this paper but will be reported in forthcoming LOCO-RISE publications.

Implications for practice and research

There is likely to be an ongoing need for community rehabilitation to support the needs of PwLC. It is clear that a 'one-size-fits-all' model of service delivery is unlikely to be desirable in geographically diverse healthcare environments, such as that found in Scotland. The impact of geographical setting was an observably limiting factor on service delivery, as more remote communities had fewer available rehabilitation staff and those that worked in remote rural communities often worked in isolation. However, the issue of rurality was confounded by included health boards with large remote rural populations also providing an integrated service delivery model of Long COVID rehabilitation. Despite this, our analysis of the data presented in this paper suggests the issue of rurality may be less pivotal than whether health boards decide to integrate Long COVID rehabilitation into existing services or develop new dedicated Long COVID rehabilitation services. Regardless of which approach is taken, this study has found several factors that require to be addressed to enable optimal Long COVID rehabilitation service delivery: proactive provision and promotion of clear referral pathways so both PwLC and primary care clinicians know of their existence and how to refer to them; sufficient staffing and range of multidisciplinary expertise to

For peer review only - http://bmjopen.bmj.com/site/about/guidelines.xhtml

meet the broad range of rehabilitation needs that PwLC require; where Health Boards provide services in remote and rural areas, AHP staff should be enabled to access professional information and specific peer support from clinicians within and outwith their own Health Board area who have greater experience of and confidence in providing rehabilitation to PwLC. This study represents the first stage in exploring community rehabilitation for Long COVID. We have also explored the perceptions of GPs and adults with Long COVID who have tried to access rehabilitation services (to be reported elsewhere). This paper is part of LOCO-RISE, a wider realist evaluation of Long COVID rehabilitation services in the four health boards in Scotland. Future papers will present further data from this evaluation. Collectively these papers will inform contextualised, evidence-based recommendations for future Long COVID rehabilitation services in Scotland. The transferability of key findings from this Scottish-based study should be considered when considering their implications for different clinical and policy contexts.

CONCLUSION

The organisational delivery of Long COVID community rehabilitation is complex and faces several challenges. In this first of a series of outputs from our LOCO-RISE study we have provided a detailed understanding of the barriers and facilitators to Long COVID community rehabilitation from the perspectives of patients, AHPs, and Long COVID leads. The knowledge presented here can be used by those developing and delivering services for people with Long COVID. Community rehabilitation services have much to offer people with Long COVID, but their optimal delivery requires adequate planning, publicity, and resource.

Author contributions

KC, ED, JM, LA, JP, JO, AL contributed to the study's conception and design. TT, VB, JC, RM, JS undertook data collection. ED, KC, TT, JC, JS, ES, RM undertook analyses and drafted the first version of the manuscript. ED led future iterations of the manuscript. All authors read and commented on the manuscript and approved the final version of it. The corresponding author attests that all authors meet authorship criteria and that nobody meeting the criteria have been omitted. ED and KC have joint responsibility for the research conduct of the study, had access to the data, and controlled the decision to publish.

Competing interests

All authors have completed the ICMJE uniform disclosure form at www.icmje.org/coi_disclosure.pdf and declare: all authors had financial support from the Chief Scientist Office Scotland (grant number COV/LTE/20/29) for the submitted work; ED has received research grants from NIHR and Scottish

For peer review only - http://bmjopen.bmj.com/site/about/guidelines.xhtml

Government for the following studies: Caring for Long COVID in primary care and DBI COVID study, respectively; no other relationships or activities that could appear to have influenced the submitted work.

Funding statement

This work was supported by the Chief Scientist Office Scotland, grant number COV/LTE/20/29

Data availability

All data produced in the present study are available upon reasonable request. Robert Gordon University holds the copyright for the full interview transcripts and may grant data sharing permission on request.

Ethics approval

This study involved human participants. Ethical approval was granted from the Wales Research Ethics Committee 6 [21/WA/0118], and each of the four health boards granted R&D management approval.

Acknowledgement

We are grateful to all our participants who gave their time to take part in interviews and to the local primary investigators from the health boards; Lynne Frew, Lynn Morrison and Gail Thomson-Patel, for their contributions to the LOCO-RISE study. We extend our sincere gratitude to the rehabilitation and administrative staff for supporting recruitment and data collection, and to Valery Burnett for her assistance with data collection.

For peer review only

REFERENCES

1. NICE SIGN RCGP. COVID-19 rapid guideline: managing the longterm effects of COVID-19 2022 [updated 28 February 2023. Available from: https://www.nice.org.uk/guidance/ng188/resources/covid19-rapid-guideline-managing-the-longterm-effects-of-covid19-pdf-51035515742202.
2. Davis HE, McCorkell L, Vogel JM, et al. Long COVID: major findings, mechanisms and recommendations. *Nat Rev Microbiol* 2023;21(3):133-46. doi: 10.1038/s41579-022-00846-2 [published Online First: 20230113]
3. Office for National Statistics (ONS). released 2 February 2023, ONS website, statistical bulletin, Prevalence of ongoing symptoms following coronavirus (COVID-19) infection in the UK 2023 [Available from: https://www.ons.gov.uk/peoplepopulationandcommunity/healthandsocialcare/conditionsanddiseases/bulletins/prevalenceofongoingsymptomsfollowingcoronaviruscovid19infectionintheuk/2february2023 accessed 28 Feburary 2023 2023.
4. NHS England. NHS sets out long COVID action plan for thousands of people with persistent symptom 2022 [Available from: https://www.england.nhs.uk/2022/07/nhs-sets-out-long-covid-action-plan-for-thousands-of-people-with-persistent-symptom/ accessed 20 July 2023 2023.
5. Welsh Government. Funding boost to open long COVID services up to people with long term conditions 2023 [Available from: https://www.gov.wales/funding-boost-open-long-covid-services-people-long-term-conditions accessed 20 July 2023 2023.
6. Scottish Government. Long COVID Support Fund 2021 [Available from: https://www.gov.scot/news/long-covid-support-fund/ accessed 20 July 2023 2023.
7. World Health Organization. Clinical management of COVID-19: living guideline, 13 January 2023. Geneva: World Health Organization, 2023.
8. DeMars J, Brown DA, Angelidis I, et al. What is Safe Long COVID Rehabilitation? *J Occup Rehabil* 2023;33(2):227-30. doi: 10.1007/s10926-022-10075-2
9. NHS England. National commissioning guidance for post COVID services 2022 [Available from: https://www.england.nhs.uk/publication/national-commissioning-guidance-for-post-covid-services/ accessed 28 February 2023 2023.
10. Buttery S, Philip KEJ, Williams P, et al. Patient symptoms and experience following COVID-19: results from a UK-wide survey. *BMJ Open Respir Res* 2021;8(1) doi: 10.1136/bmjresp-2021-001075
11. Baz SA, Fang C, Carpentieri JD, et al. 'I don't know what to do or where to go'. Experiences of accessing healthcare support from the perspectives of people living with Long Covid and healthcare professionals: A qualitative study in Bradford, UK. *Health Expect* 2023;26(1):542-54. doi: 10.1111/hex.13687 [published Online First: 20221213]
12. Macpherson K, Cooper K, Harbour J, et al. Experiences of living with long COVID and of accessing healthcare services: a qualitative systematic review. *BMJ Open* 2022;12(1):e050979. doi: 10.1136/bmjopen-2021-050979 [published Online First: 20220111]
13. Duncan E, Cooper K, Cowie J, et al. A national survey of community rehabilitation service provision for people with long Covid in Scotland. *F1000Res* 2020;9:1416. doi: 10.12688/f1000research.27894.2 [published Online First: 20201207]

For peer review only - http://bmjopen.bmj.com/site/about/guidelines.xhtml

14. Decary S, De Groote W, Arienti C, et al. Scoping review of rehabilitation care models for post COVID-19 condition. *Bull World Health Organ* 2022;100(11):676-88. doi: 10.2471/BLT.22.288105 [published Online First: 20221003]
15. Flannery T, Brady-Sawant H, Tarrant R, et al. A Mixed-Methods Evaluation of a Virtual Rehabilitation Program for Self-Management in Post-COVID-19 Syndrome (Long COVID). *Int J Environ Res Public Health* 2022;19(19) doi: 10.3390/ijerph191912680 [published Online First: 20221004]
16. Killingback C, Thompson M, Nettleton M, et al. Telerehabilitation for patients who have been hospitalised with covid-19: a qualitative study. *Disabil Rehabil* 2023:1-9. doi: 10.1080/09638288.2022.2159075 [published Online First: 20230111]
17. Parkin A, Davison J, Tarrant R, et al. A Multidisciplinary NHS COVID-19 Service to Manage Post-COVID-19 Syndrome in the Community. *J Prim Care Community Health* 2021;12:21501327211010994. doi: 10.1177/21501327211010994
18. Lloyd-Evans PHI, Baldwin MM, Daynes E, et al. Early experiences of the Your COVID Recovery((R)) digital programme for individuals with long COVID. *BMJ Open Respir Res* 2022;9(1) doi: 10.1136/bmjresp-2022-001237
19 Willis DG, Sullivan-Bolyai S, Knafl K, et al. Distinguishing Features and Similarities Between Descriptive Phenomenological and Qualitative Description Research. *West J Nurs Res* 2016;38(9):1185-204. doi: 10.1177/0193945916645499 [published Online First: 20160422]
20. Tong A, Sainsbury P, Craig J. Consolidated criteria for reporting qualitative research (COREQ): a 32-item checklist for interviews and focus groups. *Int J Qual Health Care* 2007;19(6):349-57. doi: 10.1093/intqhc/mzm042 [published Online First: 20070914]
21. Hoffmann TC, Glasziou PP, Boutron I, et al. Better reporting of interventions: template for intervention description and replication (TIDieR) checklist and guide. *BMJ* 2014;348:g1687. doi: 10.1136/bmj.g1687 [published Online First: 20140307]
22. Keith RE, Crosson JC, O'Malley AS, et al. Using the Consolidated Framework for Implementation Research (CFIR) to produce actionable findings: a rapid-cycle evaluation approach to improving implementation. *Implement Sci* 2017;12(1):15. doi: 10.1186/s13012-017-0550-7 [published Online First: 20170210]
23. Gale NK, Heath G, Cameron E, et al. Using the framework method for the analysis of qualitative data in multi-disciplinary health research. *BMC Med Res Methodol* 2013;13:117. doi: 10.1186/1471-2288-13-117 [published Online First: 20130918]
24. Goldsmith LJ. Using Framework Analysis in Applied Qualitative Research. 2021
25. Malterud K, Siersma VD, Guassora AD. Sample Size in Qualitative Interview Studies: Guided by Information Power. *Qual Health Res* 2016;26(13):1753-60. doi: 10.1177/1049732315617444 [published Online First: 20160710]
26. Brennan A, Broughan J, McCombe G, et al. Enhancing the management of long COVID in general practice: a scoping review. *BJGP Open* 2022;6(3) doi: 10.3399/BJGPO.2021.0178 [published Online First: 20220928]
27. Kingstone T, Taylor AK, O'Donnell CA, et al. Finding the 'right' GP: a qualitative study of the experiences of people with long-COVID. *BJGP Open* 2020;4(5) doi: 10.3399/bjgpopen20X101143 [published Online First: 20201215]
28. Jesus TS, Landry MD, Dussault G, et al. Human resources for health (and rehabilitation): Six Rehab-Workforce Challenges for the century. *Hum Resour Health* 2017;15(1):8. doi: 10.1186/s12960-017-0182-7 [published Online First: 20170123]

For peer review only

For peer review only - http://bmjopen.bmj.com/site/about/guidelines.xhtml

29. Gokani SA, Ta NH, Espehana A, et al. The growing burden of long COVID in the United Kingdom: Insights from the UK Coronavirus Infection Survey. *Int Forum Allergy Rhinol* 2022 doi: 10.1002/alr.23103 [published Online First: 20221208]
30. Ceban F, Leber A, Jawad MY, et al. Registered clinical trials investigating treatment of long COVID: a scoping review and recommendations for research. *Infect Dis (Lond)* 2022;54(7):467-77. doi: 10.1080/23744235.2022.2043560 [published Online First: 20220314]
31. Nguyen NN, Hoang VT, Dao TL, et al. Clinical patterns of somatic symptoms in patients suffering from post-acute long COVID: a systematic review. *Eur J Clin Microbiol Infect Dis* 2022;41(4):515-45. doi: 10.1007/s10096-022-04417-4 [published Online First: 20220210]
32. Ostrowska M, Rzepka-Cholasinska A, Pietrzykowski L, et al. Effects of Multidisciplinary Rehabilitation Program in Patients with Long COVID-19: Post-COVID-19 Rehabilitation (PCR SIRIO 8) Study. *J Clin Med* 2023;12(2) doi: 10.3390/jcm12020420 [published Online First: 20230104]
33. O'Hare AM, Vig EK, Iwashyna TJ, et al. Complexity and Challenges of the Clinical Diagnosis and Management of Long COVID. *JAMA Netw Open* 2022;5(11):e2240332. doi: 10.1001/jamanetworkopen.2022.40332 [published Online First: 20221101]
34. Noble H, Heale R. Triangulation in research, with examples. *Evidence-Based Nursing* 2019;22:67-68. doi: 10.1136/ebnurs-2019-103145
35. O'Cathain A, Murphy E, Nicholl J. Multidisciplinary, Interdisciplinary, or Dysfunctional? Team Working in Mixed-Methods Research. *Qualitative Health Research* 2008;18(11):1574-85. doi: 10.1177/1049732308325535

For peer review only

For peer review only - http://bmjopen.bmj.com/site/about/guidelines.xhtml

Consolidated criteria for reporting qualitative studies (COREQ): 32-item checklist

Please indicate in which section each item has been reported in your manuscript. If you do not feel an item applies to your manuscript, please enter N/A.

For further information about the COREQ guidelines, please see Tong *et al.*, 2017: https://doi.org/10.1093/intqhc/mzm042

No.	Item	Description	Section #
**Domain 1: Research team and reflexivity**			
Personal characteristics			
1.	Interviewer/facilitator	Which author/s conducted the interview or focus group?	
2.	Credentials	What were the researcher's credentials? *E.g. PhD, MD*	
3.	Occupation	What was their occupation at the time of the study?	
4.	Gender	Was the researcher male or female?	
5.	Experience and training	What experience or training did the researcher have?	
Relationship with participants			
6.	Relationship established	Was a relationship established prior to study commencement?	
7.	Participant knowledge of the interviewer	What did the participants know about the researcher? *E.g. Personal goals, reasons for doing the research*	
8.	Interviewer characteristics	What characteristics were reported about the interviewer/facilitator? *E.g. Bias, assumptions, reasons and interests in the research topic*	
**Domain 2: Study design**			
Theoretical framework			
9.	Methodological orientation and theory	What methodological orientation was stated to underpin the study? *E.g. grounded theory, discourse analysis, ethnography, phenomenology, content analysis*	
Participant selection			
10.	Sampling	How were participants selected? *E.g. purposive, convenience, consecutive, snowball*	
11.	Method of approach	How were participants approached? *E.g. face-to-face, telephone, mail, email*	
12.	Sample size	How many participants were in the study?	
13.	Non-participation	How many people refused to participate or dropped out? What were the reasons for this?	
Setting			
14.	Setting of data collection	Where was the data collected? *E.g. home, clinic, workplace*	
15.	Presence of non-participants	Was anyone else present besides the participants and researchers?	

For peer review only

16.	Description of sample	What are the important characteristics of the sample? *E.g. demographic data, date*	
Data collection			
17.	Interview guide	Were questions, prompts, guides provided by the authors? Was it pilot tested?	
18.	Repeat interviews	Were repeat interviews carried out? If yes, how many?	
19.	Audio/visual recording	Did the research use audio or visual recording to collect the data?	
20.	Field notes	Were field notes made during and/or after the interview or focus group?	
21.	Duration	What was the duration of the interviews or focus group?	
22.	Data saturation	Was data saturation discussed?	
23.	Transcripts returned	Were transcripts returned to participants for comment and/or correction?	
**Domain 3: analysis and findings**			
Data analysis			
24.	Number of data coders	How many data coders coded the data?	
25.	Description of the coding tree	Did authors provide a description of the coding tree?	
26.	Derivation of themes	Were themes identified in advance or derived from the data?	
27.	Software	What software, if applicable, was used to manage the data?	
28.	Participant checking	Did participants provide feedback on the findings?	
Reporting			
29.	Quotations presented	Were participant quotations presented to illustrate the themes / findings? Was each quotation identified? *E.g. Participant number*	
30.	Data and findings consistent	Was there consistency between the data presented and the findings?	
31.	Clarity of major themes	Were major themes clearly presented in the findings?	
32.	Clarity of minor themes	Is there a description of diverse cases or discussion of minor themes?	

When submitting your manuscript via the online submission form, please upload the completed checklist as a Figure/supplementary file.

If you would like this checklist to be included alongside your article, we ask that you upload the completed checklist to an online repository and include the guideline type, name of the repository, DOI and license in the *Data availability* section of your manuscript.

Developed from: Allison Tong, Peter Sainsbury, Jonathan Craig, Consolidated criteria for reporting qualitative research (COREQ): a 32-item checklist for interviews and focus groups, International Journal for Quality in Health Care, Volume 19, Issue 6, December 2007, Pages 349–357, https://doi.org/10.1093/intqhc/mzm042

For peer review only

SUPPLEMENT File 1: Interview Topic Guides

Explanation of Supplement File 1

This file contains three interview topic guides, which were tailored for the three groups of study participants: 1) Long COVID leads; 2) Staff Delivering long COVID rehabilitation services (AHPs); and 3) People with long COVID (PwLC). The first guide, for long COVID leads, was informed by the Consolidated Framework for Implementation Research (CFIR). The CFIR categories are noted in blue.

Long COVID leads and AHPs were interviewed multiple times, whereas PwLC were interviewed at one time point. The second guide, for Staff Delivering long COVID rehabilitation, includes reframed questions in red for the second interview.

Purpose

This topic guide supports the data collection to explore NHS Heath Board senior management perceptions of how decisions are made regarding the allocation of Scottish Government funding for Long COVID and in particular how decisions were/are made regarding funding, model of service provision and publicity for long COVID rehabilitation in community settings.

The topic guide is deliberately broad and more specific areas for questioning will be driven by the topics raised during the interview. You do not have to ask questions in the order they are presented but should use the questions as a checklist to ensure all key topic areas are covered.

1. Interview Topic Guide for Long COVID Leads

SECTION 1: GENERAL INTRODUCTIONS

1.1 General introduction by interviewer to the purpose of the interview (Researcher note: relay purpose of interview as described above above).

1.2 General introduction by the interviewee of their managerial role in general and specifically in relation to long COVID.

1.2.1 Please could you tell me about your role within [name NHS Health Board]?

1.2.2 Specifically, could you tell me what role you have in the decision-making process regarding the Health Board's decision regarding delivery of long COVID? **(Process)**

1.2.3 If they do not mention their role in funding decisions relating to long COVID services probe for that.

SECTION 2: SERVICE DELIVERY

2.1 Can you describe to me how long COVID services have been provided to date by [name NHS Health Board]? (Service characteristics)

For peer review only - http://bmjopen.bmj.com/site/about/guidelines.xhtml

2.1.1 What are the services that are delivered for long COVID within [NHS Health Board]? (Service characteristics)

2.1.2 What were the drivers for introducing these services and the way in which they were/are delivered (internal {e.g. within Health Board}/external {political, societal etc})? **(Inner/Outer setting; Process)**

2.1.1 How were these services funded? **(Inner/Outer settings)**

2.1.2 Have long COVID services changed at any point within your service? [If yes, then probe regarding why they changed and in what way]. **(Service characteristics/ Inner/Outer setting)**

2.1.2 What are/were the challenges that [NHS Health Board] face(d) in delivering long COVID services? **(Inner/Outer settings; Process)**

2.3 How have your services changed (if at all) since the arrival of COVID?

2.3.1 In what way/if any/ has [NHS Health Board] promoted these services to key referrer agencies (such as GPs and others in Primary Care), and the public? What has guided the decisions you have made re promoting long COVID services to Primary Care, the community and other stakeholders?

2.3. 1 What is your general attitude towards this model of community rehabilitation? (How has it been going)? [Prompts: positive/beneficial – please elaborate, negative/detrimental – please elaborate, unclear etc.]

2.2 On 30 September 2021 the Scottish Government announced additional funding of £10 million pounds for long COVID services. How has that allocation been decided for NHS Health Boards? What share did [NHS Health Board] receive? (Inner/Outer settings; Processes)

2.2.1 Within [NHS Health Board] what was the decision-making process for how that money would be spent? **(Inner setting; Processes)**

2.2.2. Will rehabilitation services for people with long COVID in the community benefit from this funding? If yes, then how? If no, then why was spending not priorities for this? **(Intervention; Processes; Inner setting)**

2.2.3 If there will be new services, how will these new services be communicated to Primary Care and the public and other relevant stakeholders?

SECTION 3: FACILITATORS AND BARRIERS

3.1 What (if anything) do you think is working well about how long COVID services – and particularly rehabilitation for people with long COVID are being delivered for people in [NHS Health Board].

3.2.1 What has enabled this/these to work well?

3.2 What (if any) barriers do you feel there are in delivering high quality services for people with long COVID, in particular that requiring long COVID rehabilitation? How might these barriers be overcome?

For peer review only - http://bmjopen.bmj.com/site/about/guidelines.xhtml

SECTION 4: THE FUTURE

4.2 How do you see long COVID services being delivered over the coming months and years? (Intervention; Outer/Inner Setting; Process)

4.1 How do you foresee long COVID rehabilitation in [NHS Health Board] being delivered over the coming months and years?

4.1.1 What are the funding implications?

SECTION 5: CLOSING THE INTERVIEW

5.1 Is there anything else we have not covered regarding funding and delivery of long COVID, particularly in relation to rehabilitation in the community?

Notes for interviewer

- Check topic guide and return to any questions as necessary
- If no further questions/discussion remains, thank participant for their time
- Save/stop the recording
- Note down any interviewer/participant observations
- Upload recording to CSO R Drive (RGU) and include any notes taken

For peer review only

For peer review only - http://bmjopen.bmj.com/site/about/guidelines.xhtml

2. Interview Topic Guide for Staff Delivering Long COVID Rehabilitation Services

Aim

To explore staff perceptions of how community rehabilitation for people experiencing symptoms of long COVID is being delivered. To explore their perception of the barriers (and how these could be overcome) and facilitators (and how these could be optimised) for success. To explore their perception of the impact of community rehabilitation on patient outcomes for this population. The interviews have a general purpose to obtain feedback from those involved in delivering community rehabilitation for people with long COVID.

The specific aim of follow-up interviews is to get a sense of what, if anything has changed in the last few months. This could be at an organisational level, or at an individual professional level in relation to long COVID.

(AHPs)

Remember – we want you to have a good conversation with people about community rehabilitation for people with long COVID. The questions are there to provide a bit of structure and to make sure you cover all required basis, but it is not an interview schedule and you do not have to follow them in order.

Tasks at or before start of interview:

(Re)Introduce self and relax participant through welcoming conversation

Check participant has read participant information sheet and understood aims and consents

Assign code to each new participant (e.g. L001 for Lanarkshire participant). Use previous code if a repeat interview assigning the letter b (if 2nd interview).

Participant information to be gathered at start of each interview: profession, gender, years qualified, Agenda for Change band (Ask if what 'Band' they are; this is their professional grade), length of time they have worked in community rehabilitation. Check that details have stayed the same since last interview if it is a repeat interview. If not, note new details.

SECTION 1: GETTING STARTED

Can you describe your role within the community rehabilitation team?

How has this role been for the last few months? Have there been any changes in relation to long COVID patients?

In this role, have you had any seen any/many patients who are experiencing long COVID?

(if they have seen long COVID patients) Can you tell me about what your role has been with patients with long COVID? Has this changed at all over the last few months?

For peer review only - http://bmjopen.bmj.com/site/about/guidelines.xhtml

(if they have seen long COVID patients) How confident have you been in delivering therapy for people with long COVID symptoms? Has this changed at all over the last few months?

(if not seen any) Have you been aware of any of your colleagues treating people due to problems related to long COVID? What have they done? Has this changed at all over the last few months?

SECTION 2: SERVICE DELIVERY

(Covers TiDIER criteria – Why, What Who, How, Where, When & How much, Tailoring)

Tell me about rehabilitation for patients with long COVID within your team/department?

Have there been any changes to how long COVID is delivered in your service? If so, can you describe these changes?

- How are people referred to your service? Who by? How do you let people know about your service?
- Why are people being referred for long COVID rehabilitation?
  - What problems? What are the general goals of rehabilitation for the people that have been seen?
- What happens after you received a referral (triage? Who decides who sees the person? How is initial contact (telephone/online/f2f)
- What happens after initial contact?
- What rehab services are offered?
  - e.g. assessment & advice only/self-management & signposting/set number of sessions/individual to patient needs.
  - How is that process decided?
  - Who provides rehabilitation? How is it decided who sees each patient?
- Where forms of therapy are generally delivered?
  - what do the professionals do?
- Typically, how intense is the rehabilitation process?
  - frequency/duration of interventions, how long on caseload?
- Is this different or the same as other patients accessing your service/department?
- In what way is the therapy tailored to the individual being treated?
- How do you decide when to stop intervention?

SECTION 3: FACILITATORS AND BARRIERS

What is your perception of the potential of community rehabilitation for people with long COVID?

Has this changed at all over the last few months?

- What seems to work well for people who have long COVID? (Consider: engagement, outcomes, access to services, development of service etc.)
  - Why/in what way?

For peer review only - http://bmjopen.bmj.com/site/about/guidelines.xhtml

- What doesn't work so well in delivering community rehabilitation for people with long COVID (Consider: referral process, mode of therapy delivery, duration of intervention, admin, resources etc.)
    - Why/how could these be overcome?

SECTION 4: THE FUTURE

How could services change to better suit needs of patients with long COVID who need rehabilitation?

What do you see as the big challenges facing long COVID rehabilitation just now?

- Consider: staffing: funding, mode of delivery, any services not currently provided, referral to other services, central long COVID clinics vs integrated services within existing structures.

SECTION 5: CLOSING THE INTERVIEW

Is there anything else we have not talked about but you think would be beneficial in our understanding of community rehabilitation for people with long COVID?

Notes for interviewer:

- Check topic guide that all key areas are covered
- Thank participant for their time
- Save and stop recording
- Note down any participant observations or thoughts re the interview
- Upload recording to CSOR Drive (RGU) with any notes taken

For peer review only

3. Interview Guide for People with Long COVID (PwLC)

Aim

To obtain input from people living with long COVID, their perceptions of the acceptability of the community rehabilitation service, their preferences for service delivery models and what a responsive service might look like (referral, location, duration, modes of delivery etc…).

Interviews have a general purpose to obtain ideas from participants about what an ideal community rehabilitation service for people living with long COVID might look like, what they perceive to be the barriers and facilitators to the model they are experiencing and what gaps, if any, they feel exist in service delivery. Therefore, the topic guide is deliberately broad and more specific areas for questioning will be driven by the questions and interests of the people taking part.

Welcome and introductions.

Check they can hear ok.

Thank you for agreeing to meet with me. Introduce self to participant.

I hopefully won't take too much your time up.

Structure of call.

Could I just start by going though the verbal consent, just to make sure that you are happy with everything? Inform them that we will go through the consent process which asks a few statements and if you can say yes/no that would be great. Just so you know that the interview will be audio recorded just so we can refer back to the data if that's ok with you.

I have 8 statements that I will read and if you are happy, if you can just say yes at the end of them that would be great.

Ask to say name. <CONSENT>

Great, thank you for doing that – it just keeps us all right. I will stop the recording there.

Opportunities for the participant to ask questions about the study and to confirm verbally that they are still happy to participate.

Re-start new recording

Opening statement:

Thank you again for taking the time to speak to me. And just to give you a bit of background about the study. Essentially, we're doing a project over two years just looking at long COVID rehabilitation across Scotland. We're looking across four Scottish health boards. And as you can understand we are still learning about long COVID and things are changing very quickly. So, we are kind of trying to see how things are changing over these two years right from start in relations to rehabilitation service. What we've been doing is speaking to staff that deliver the service across Scotland also sort of managers and directors at each of those health boards. But we also want to hear from people like yourselves and who have experienced COVID and experiencing long COVID, just in terms of getting

For peer review only - http://bmjopen.bmj.com/site/about/guidelines.xhtml

your viewpoints about your experiences, what's been good, what's been bad and so forth. We are really interested to hear things from your side of things if that's alright?

Start Interview questions

These can be asked in any order and are there to guide discussion. Feel free to use your own words to ask the questions in as open a manner as possible. What is important is the topics are covered.

Q1. Can you tell me a bit about your COVID story?

Prompts: When were you diagnosed? Was it a formal diagnosis following a test or did you feel you matched the symptoms? When did you begin to consider you had long COVID? How is COVID affecting you now?

Prompts:
Job: What is your job now?
Symptoms mentioned? Any other symptoms?
Did you seek any help from GP? What did the GP do? Refer to other services? What services?
And how did that work for you?
Has that changed? Have you had any face-to-face appointments or is it just stayed virtual, online?
Anything you thought could be better? What do you think went well for you?

Q2. How did you get referred for long COVID rehabilitation?

Prompts: Who referred you?
What did they refer you for (e.g. fatigue, breathlessness, brain-fog etc.),
was it easy to get referred?
How long did they wait to be seen?

Q3. For how long and how often have you been receiving long COVID rehabilitation (weeks and sessions [**when and how much]**)?

Q4. What had you hoped/do you hope to achieve from community rehabilitation?

Q5. Can you tell me a bit about the rehabilitation you have received to date?

Prompts: **Who** has provided your long COVID rehab (i.e. what type of professionals – physiotherapists, occupational therapists etc);
What has your rehabilitation consisted of so far (i.e. what procedures or advice, or other activities); **How** was it provided? (e.g. face to face or in a group, online or telephone);
Where was it provided? (if face to face was it at home or in a clinic etc)

Q6. What are your experiences of long COVID rehabilitation to date?

Prompts: Do you feel the service is/was responsive to your needs?
How do you feel about the number of times and frequency with which you were seen?
How appropriate do you think the service's way of delivering rehabilitation has been/is (e.g. face to face/digital/self-management) for you?

Q7. Are there any particular aspects that you think are working well or lessons learned to improve long COVID rehabilitation you are receiving?

Prompt: What is working well?
What could be better? (Note – try and tap into service issues such as referral, delivery mode etc, as well as individual issues)

For peer review only - http://bmjopen.bmj.com/site/about/guidelines.xhtml

Q8. Is there anything else you would like to share about your experience of the community rehabilitation service?

Closing statement:

That’s all the questions I’ve got. Is there anything else that we've not touched on that you think you know important to mention.

Great. Well, thank you again. Lovely to meet you and thank you. As you said, you're happy enough if there's anything else in the future, if you're happy for me to get back in touch.

I was going to ask if there's anything in the future that I think of, would you be happy for me to contact you again just via email or something and see how you're getting on?

Thanks, and conclude interview.

Turn off recorder

For peer review only

For peer review only - http://bmjopen.bmj.com/site/about/guidelines.xhtml

Investigating Scottish Long COVID community rehabilitation service models from the perspectives of people living with Long COVID and healthcare professionals: a qualitative descriptive study

Journal:	*BMJ Open*
Manuscript ID	bmjopen-2023-078740.R2
Article Type:	Original research
Date Submitted by the Author:	13-Nov-2023
Complete List of Authors:	Duncan, Edward; University of Stirling, NMAHP-RU<br>Alexander, L; Scottish Centre for Evidence-Based Multi-Professional Practice; Robert Gordon University, School of Health Sciences<br>Cowie, Julie; Glasgow Caledonian University, NMAHP Research Unit<br>Love, Alison; Robert Gordon University<br>Morris, Jacqui H.; University of Dundee, SNHS<br>Moss, Rachel; Healthcare Improvement Scotland<br>Ormerod, Jane; Robert Gordon University<br>Preston, Jenny; Ayrshire Central Hospital, Douglas Grant Rehabilitation Centre<br>Shim, Joanna; Robert Gordon University, School of Health Sciences<br>Stage, Emma; Robert Gordon University, School of Health Sciences<br>Tooman, Tricia; University of Stirling, NMAHP-RU<br>Cooper, K; Robert Gordon University, School of Health Sciences
**Primary Subject Heading**:	Rehabilitation medicine
Secondary Subject Heading:	Evidence based practice
Keywords:	Post-Acute COVID-19 Syndrome, HEALTH SERVICES ADMINISTRATION & MANAGEMENT, REHABILITATION MEDICINE

SCHOLARONE™
Manuscripts

I, the Submitting Author has the right to grant and does grant on behalf of all authors of the Work (as defined in the below author licence), an exclusive licence and/or a non-exclusive licence for contributions from authors who are: i) UK Crown employees; ii) where BMJ has agreed a CC-BY licence shall apply, and/or iii) in accordance with the terms applicable for US Federal Government officers or employees acting as part of their official duties; on a worldwide, perpetual, irrevocable, royalty-free basis to BMJ Publishing Group Ltd ("BMJ") its licensees and where the relevant Journal is co-owned by BMJ to the co-owners of the Journal, to publish the Work in this journal and any other BMJ products and to exploit all rights, as set out in our licence.

The Submitting Author accepts and understands that any supply made under these terms is made by BMJ to the Submitting Author unless you are acting as an employee on behalf of your employer or a postgraduate student of an affiliated institution which is paying any applicable article publishing charge ("APC") for Open Access articles. Where the Submitting Author wishes to make the Work available on an Open Access basis (and intends to pay the relevant APC), the terms of reuse of such Open Access shall be governed by a Creative Commons licence – details of these licences and which Creative Commons licence will apply to this Work are set out in our licence referred to above.

Other than as permitted in any relevant BMJ Author's Self Archiving Policies, I confirm this Work has not been accepted for publication elsewhere, is not being considered for publication elsewhere and does not duplicate material already published. I confirm all authors consent to publication of this Work and authorise the granting of this licence.

For peer review only

TITLE: Investigating Scottish Long COVID community rehabilitation service models from the perspectives of people living with Long COVID and healthcare professionals: a qualitative descriptive study.

ABSTRACT

Objectives: To explore the perceptions and experiences of barriers and facilitators to accessing Long COVID community rehabilitation.

Design: We used a qualitative descriptive design over two rounds of data collection with three participant groups: i) people with experience of rehabilitation for Long COVID (PwLC); ii) NHS staff delivering and/or managing community rehabilitation services (allied health professionals (AHPs)), and iii) NHS staff involved in strategic planning around Long COVID in their health board (Long COVID leads).

Setting: Four NHS Scotland territorial health boards.

Participants: 51 interviews: eight Long COVID leads (11 interviews); 15 AHPs (25 interviews) and 15 PwLC (15 interviews).

Results: Three key themes were identified: i) Accessing care for PwLC, ii) Understanding Long COVID and its management; and iii) Strengths and limitations of existing Long COVID rehabilitation services.

Conclusions: Organisational delivery of Long COVID community rehabilitation is complex and presents multiple challenges. In addition, access to Long COVID community rehabilitation can be challenging. When accessed, these services are valued by PwLC but require adequate planning, publicity, and resource. The findings presented here can be used by those developing and delivering services for people with Long COVID.

Strengths and limitations of this study

- Studying four Health Boards enabled an analysis of development of differing service models of delivery over the same time period
- The rapidly evolving nature of Long COVID and its management resulted in fewer distinct differences between the four health boards than originally anticipated
- Study findings are limited to Scottish Long Covid rehabilitation context.
- Other relevant perspectives, such as General Practice/family medicine are not included in this study but will be presented in subsequent publications.

For peer review only - http://bmjopen.bmj.com/site/about/guidelines.xhtml

INTRODUCTION

Background

Long COVID, a condition defined as ongoing COVID-19 symptoms that continue beyond twelve weeks following an initial acute COVID-19 infection, was first described in Spring 2020 [1]. It is estimated that at least 65 million people globally have Long Covid [2]. By January 2023, over 2 million people were estimated to have Long COVID in the United Kingdom (UK), including over 175,000 living in Scotland [3]. Long COVID has broad multiple system presentation and can have profound physical, emotional, social, and financial consequences. Moreover, debilitating symptoms of Long COVID often persist for a year or more [2].

NHS funding to support the diagnosis, treatment, and rehabilitation for Long COVID varies across the UK. Since the autumn of 2020, NHS England have allocated £94 million (£1.66 per capita, based on the 2021 UK Office of National Statistics Census data England's population was 56,489,800) to be invested in specialist Long COVID clinics in England to complement existing primary, community, and rehabilitation care services [4]. The Welsh Government has allocated £18.3 million since 2020 (£5.88 per capita based on a population of 3,107,500 in 2021) to support the delivery of Long COVID diagnosis, treatment, and rehabilitation within existing services [5]. In September 2021, the Scottish Government announced £10 million (£1.82 per capita based on a population of 5,479,900 in 2021) over 3 years to support Scottish NHS health boards' response to Long COVID [6]. At the end of May 2022, plans for the first £3 million of that funding were announced.

The National Institute for Health and Care Excellence (NICE) [1] and The World Health Organisation [7] recommend people with Long COVID, following an initial medical assessment to exclude underlying conditions, have access to rehabilitation to aid their recovery. As with other long-term conditions, community rehabilitation for people with Long COVID should be personalised, multidisciplinary, and comprehensive in order to maximise function, quality of life, and participation in society [8 9].

However, the optimal approach to deliver Long COVID community rehabilitation is currently unknown. NICE clinical guidance suggests broad rehabilitation approaches, which can be delivered in isolation or in combination, such as self-management and multidisciplinary rehabilitation [1]. The effectiveness of these approaches is not yet clear and there are further uncertainties about the most appropriate means of organising service delivery, particularly that of multidisciplinary rehabilitation. There are well-documented barriers to accessing healthcare services for PwLC [10-12]; however previous research has not specifically considered rehabilitation services. Our 2019 national survey [13] of Scotland's 14 territorial health boards found that Long COVID community rehabilitation was universally available across Scotland. At the time of the survey, one health board

For peer review only - http://bmjopen.bmj.com/site/about/guidelines.xhtml

was delivering Long COVID community rehabilitation as a dedicated service, with the remainder integrating rehabilitation within existing services. And yet, there was substantial variation across health boards in the mode of service delivery, given that the means of optimally delivering community rehabilitation to this population was unknown.

To date rehabilitation services for Long COVID have varied internationally with respect to delivery and the healthcare professionals involved [14], including virtual rehabilitation focusing on self-management and education [15], group-based pulmonary rehabilitation [16], tiered multidisciplinary pathways [17], and phased approaches to managing symptoms [18]. Our research group are investigating models of Long COVID rehabilitation service delivery in an overarching study called LOCO-RISE. LOCO-RISE employed a realist evaluation design to investigate different service delivery models for Long COVID community rehabilitation in Scotland between November 2021 – July 2023. LOCO-RISE aims to provide responsive evidence-based recommendations to the NHS about how to most effectively deliver community rehabilitation services for people with Long COVID. The aim of the phase of LOCO-RISE reported in this paper was to explore key stakeholders perceptions and experiences of the barriers and facilitators to implementing Long COVID community rehabilitation.

METHODS

Study Design

This paper reports on the first two rounds of data collection, which took place over a 12 month period. We employed a longitudinal qualitative descriptive [19] research design based on 51 interviews with three key participant groups, each of which provided distinct perspectives; i) people with experience of rehabilitation for Long COVID (PwLC), who provided lived experiences of receiving Long Covid rehabilitation; ii) NHS staff who provided perspectives of delivering and/or managing community rehabilitation services for PwLC (allied health professionals (AHPs)), and iii) NHS staff involved in strategic planning around Long COVID in their health board (Long COVID leads) who provided broader organisational perspectives. The study is reported in accordance with the Consolidated criteria for reporting qualitative research [COREQ] [Supplemental File 1])[20]. Qualitative description was selected over other qualitative designs as it enabled the exploration of a range of shared experiences to generate a rich description [19] of barriers and facilitators to the implementation of Long COVID rehabilitation.

For peer review only - http://bmjopen.bmj.com/site/about/guidelines.xhtml

Setting

The study was conducted in four Scottish health boards (HB1-HB4). The boards were selected for their geographical and demographic spread as well as variation in Long COVID rehabilitation service models, summarised in Table 1. As shown in Table 1, all boards adopted some form of multi-disciplinary team (MDT) approach using a blended mode of delivery (face-to-face, telephone, Near Me). Staffing varied; occupational therapy and physiotherapy were most consistent across services, with some including additional professions such as dietetics, speech and language therapy, and psychology. Some staff were co-located, while others worked as part of a team, but geographically remote. Notably, none of the services included medical staff.

For peer review only

Table 1: Description of participating health boards

**Health Board**	**Health Board 1**	**Health Board 2**	**Health Board 3**	**Health Board 4**
**Setting**	Largely rural	Large urban/rural area	Largely urban	Large urban/rural area
**Round 1 Long COVID service delivery model**	Integrated service: community rapid response rehabilitation	Integrated service: community rehabilitation	Integrated service: community rehabilitation	Dedicated long COVID service
	**MDT:** Physiotherapy, Occupational Therapy, Nursing, Community Psychiatric Nurse (CPN), Pharmacy, Advanced Nurse Practitioner (ANP), and Speech and Language Therapy, with links to GP and Dietetics	**MDT:** Physiotherapy, Occupational Therapy, Dietetics, and Speech and Language Therapy.	**MDT:** Physiotherapy, Occupational Therapy, Psychology, Speech and Language Therapy, and Rehabilitation Assistant Practitioner, Physiotherapy, Occupational Therapy, Psychology, Dietetics, and Speech and Language Therapy	**MDT:** Physiotherapy, Occupational Therapy, Psychology, Dietetics, and Speech and Language Therapy
	Blended delivery	Blended delivery with centralised point of referral	Blended delivery	Blended delivery
**Round 2 Long COVID service delivery model**	As Round 1	As Round 1	Integrated service moving towards a soft launch of dedicated long COVID Service<br><br>Blended delivery with single access point	Dedicated Long COVID Service via a 3-tiered triage system<br><br>Service temporarily halted due to large volume of referrals and subsequent decline in staffing and funding

Key: MDT=Multidisciplinary

For peer review only - http://bmjopen.bmj.com/site/about/guidelines.xhtml

Recruitment and Data Collection

Two rounds of data collection took place: Round 1 (November 2021 – April 2022) and Round 2 (June – July 2022). In each round, three participant groups took part: PwLC, AHPs, and Long COVID leads. Long COVID leads were purposively sampled by email invitation or word of mouth, for their role in leading on Long COVID within their health board, and included AHP directors, medical directors, and clinical service leads. Long COVID leads who only managed in-patient services were ineligible, since this study was exploring community rehabilitation. AHPs, most often occupational therapists and physiotherapists, were involved in providing Long COVID rehabilitation. Most AHPs acted in a team member role, though a small number were also clinical service leads. AHPs involved in the treatment of PwLC in the study health board areas were purposively recruited by email invitation. The invitation was sent by senior managers on behalf of the study team and explained the reasons for the research. Student AHPs were ineligible, as were AHPs who only provided in-patient Long COVID rehabilitation. Finally, a convenience sample of PwLC were recruited by their AHP. PwLC were provided with a study pack in printed or electronic format that explained the purpose of the study and volunteers opted into the study by contacting the research team by email, telephone, or leaving their contact details after completing a survey of health-related outcomes as part of the larger study. Patients were ineligible if they were aged under 18, required specialist (in-patient) management of Long COVID complications, had another life-limiting illness with reduced life-expectancy (e.g., disseminated malignancy) or were unable or unwilling to provide informed consent. Purposive sampling criteria were informed by prior knowledge of the emerging Long COVID services in each Health Board. We aimed to recruit 12 AHPs, 4 -8 Long COVID leads and up to 32 patients for each round of data collection. These sample sizes were based pragmatic considerations including the researchers' knowledge of each included Health Board's community rehabilitation services staffing profiles; time and financial constraints; and the challenges of recruiting PwLC whose health limiting condition was hypothesised to make participation in the research challenging. We did not seek to reach data saturation, but instead considered the 'information power' of the sample [21]. Malterud et.al's construct of 'information power' [21] argues that it is the amount of information the sample holds, rather than the size of the sample itself, that is the important factor when considering sample sufficiency. The 'information power' of the sample in this study was strengthened by the match of the sample to the study's specific aim, the specificity of the sample in relation to their experiences of Long COVID rehabilitation, the depth of the interviews being conducted and the applied nature of the investigation.

Interview topic guides [Supplemental file 2] were developed by the research team that included people with lived experience of Long COVID. Topic guides were further refined for Round 2 to take

For peer review only - http://bmjopen.bmj.com/site/about/guidelines.xhtml

account of the temporal nature of the questions being asked. The topic guides combined a theory-based approach using the TIDieR intervention description categories [22] to enable a comprehensive description of each rehabilitation service model, which can be conceived of as an organisational level intervention. Questions to long COVID leads were based on constructs from the Consolidated Framework for Implementation Research [23] to ascertain the factors that were associated with the intervention's implementation and a series of questions exploring facilitators and barriers to delivering and receiving community Long COVID rehabilitation. The interview topic guides were pilot tested with people who had lived experienced of Long Covid and with two AHPs who were not involved in the study prior to implementation. Web-based [Microsoft Teams] or telephone interviews were conducted by five experienced healthcare researchers with master's or doctoral level qualifications and experience of qualitative research [VB, JC, RM, JS, TT]. All interviewers were female and had experience of qualitative NHS data collection and analysis. Two of the researchers were AHPs and had prior awareness of the challenges of delivering rehabilitation. All interviewers were provided with training and supervision from KC and ED and had no prior relationship with the services or participants. Informed consent was obtained from all participants and recorded verbally prior to each interview. Interviews lasted between 22 and 86 minutes (average 52 minutes). Only the participant and interviewer were present during each interview. Interviews were audio recorded and transcribed intelligently by an external transcription service, where redundant words and/or sounds were removed. Field notes were taken during some interviews. These notes added to team discussions to support understanding of the data as it was collected but did not form part of the official data analysis. Transcripts were not returned to participants for comment or correction.

Data analysis

Data were analysed using the Framework method, which is commonly used in applied health research and recognised as appropriate for multidisciplinary research teams [24]. As a form of thematic analysis, the framework approach was congruent with the qualitative descriptive design being employed [19].

For each participant group, at least two researchers from the team familiarised themselves with at least 20% of transcripts, making analytical notes directly on the transcripts. The researcher pairs were from different disciplinary backgrounds (occupational therapy, physiotherapy, psychology, applied health research), thereby ensuring interpretation from a range of perspectives. Following discussion between all team members involved in familiarisation, a framework for analysis and interpretation was constructed [24]. The use of multiple researchers at this stage aimed to mitigate for bias in interpretation that may have occurred due to the disciplinary backgrounds of some team

For peer review only - http://bmjopen.bmj.com/site/about/guidelines.xhtml

members. Although line-by-line coding is common, it is also possible to develop a framework without engaging in explicit coding [25]; we adopted the latter approach. The ‘analytical framework’ [23] was applied across all interview transcripts using highlighting and the comments function in Microsoft Word, with regular review and discussion within the team. A charting matrix was created in Microsoft Excel that involved summarising broad categories of data from the transcripts, then interpreting the charted data by exploring within and between-cases to derive concepts and themes. These were then grouped into overarching themes. All researchers were involved in interpreting the data during regular team discussions. Participants did not directly provide feedback on the findings. However, the findings were presented in a webinar attended by Long Covid Leads, AHPs and PwLC, several of whom had participated in the study. The webinar attendees endorsed the study findings and reflected that they resonated with their personal experiences.

Patient and Public Involvement

Two people with lived experience of Long COVID were core members of the research team. They co-developed study materials and interview topic guides and contributed to analysis and interpretation of study findings.

RESULTS

Participant characteristics

We recruited eight Long COVID leads, who took part in 11 interviews over the two rounds of data collection: 15 AHPs (25 interviews) and 15 PwLC (15 interviews). We did not meet our recruitment target for PwLC despite a high conversion rate (invitation to take part in the study resulting in consent and participation). The low recruitment rate occurred as a result of apparent barriers to PwLC being referred to the community rehabilitation services, a phenomenon that was subsequently explored in a related study (to be reported elsewhere). See Table 2 for participant details. No participants who consented withdrew from the study.

For peer review only

Table 2 Participant demographics

	Long COVID leads (L)	Staff delivering service/AHPs (S)	Patients (P)
	**N=6**<br>**Rounds 1 & 2 N=3**<br>**Round 1 N=2**<br>**Round 2 N=1**	**N=15**<br>**Rounds 1 & 2 N=9**<br>**Round 1 N=2**<br>**Round 2 N=4**	**N=15**
**Age:**			
Working age [%]	8 [100]	15 [100]	13 [87]
		AfC Band / **N=**	
		6 / 7	
		7 / 4	
		8a / 4	
**Sex:**			
Female [%]	6 [75]	15 [100]	12 [80]
Male [%]	2 [25]	0	3 [20]
**Employment status:**			
Working full-time [%]	8 [100]	15 [100]	6 [40]
Working part-time [%]	0	0	1 [7]
Phased return to work [%]	0	0	2 [13]
Retired [%]	0	0	2 [13]
Unable to work because of illness [%]	0	0	3 [20]
Not provided [%]	0	0	1 [7]
**Time from first COVID diagnosis**	NA	NA	12 – 28 months

Key: AHPs = Allied Health Professionals; AfC = Agenda for Change

For peer review only

Findings

Framework analysis resulted in three key themes being identified: i) Accessing care for PwLC, ii) Understanding Long COVID and its management; and iii) Strengths and limitations of existing long COVID rehabilitation services. Table 3 illustrates the three themes and the categories of data that contributed to each.

Table 3: Key themes and categories of data

Key Themes	Sub themes
**Accessing care for people with Long COVID**	PwLC accessing General Practitioner (GP) services [L; P]<br>GP referring PwLC to community rehabilitation [L; P; S]<br>Awareness of services by GPs & PwLC [S]<br>Signposting [S]<br>Waiting lists & waiting times [P]
**Understanding Long COVID and its management**	Long COVID prevalence [L]<br>Long COVID unknowns [L; P]<br>Reluctance to diagnose [P]<br>Tests/investigations [P]<br>Being believed [P]<br>Evolving evidence-base [L; S]<br>Education of PwLC and community rehabilitation staff [S]<br>Information sharing [S]<br>Learning from other conditions [S]<br>Confidence of community rehabilitation staff [S]
**Long COVID services**	Current services strengths & limitations [L; P; S]<br>Resourcing services [L; P; S]<br>Need for Long COVID services [L; P; S]<br>Navigating politics [L]

Key: L = Long Covid leads; P = PwLC; S = AHPs

Participant data was coded and reported as follows: (1) Role of participant: L = Long Covid leads; P = PwLC; S = AHPs; (2) Health Board (HB) number: HBs 1-4; (3) Round of data collection: 1 or 2; and (4) Participant number (e.g. the participant identifier for the first quote: P-3-2-08 is a PwLC from HB3, data was collected in Round 2 and was the 8th PwLC interviewed).

For peer review only - http://bmjopen.bmj.com/site/about/guidelines.xhtml

I: Accessing care for People with Long COVID

Barriers to accessing Long COVID rehabilitation were identified by all participant groups. In Round 1, patients commonly attributed this to limited availability of face-to-face GP appointments due to COVID-19 restrictions.

> *"You don't get face-to-face with the GP. So again, when you get a GP, it's always a different GP you're talking to and you try your best, but you get locum, and they've all been fantastic. I'm not, again, I wouldn't criticise them, but it's exhausting telling the same story every single time." [P3208]*

Long COVID leads and AHPs observed people's reluctance to seek help from services widely acknowledged as being under pressure. By Round 2, they felt that the perception of GPs being closed, and services overwhelmed, needed to be questioned, and called for greater public awareness of Long COVID rehabilitation services and how to access them.

> *"[We] need to get past the public's perception that GPs are shut so they return to their GP and get a referral to their service if required." [L3102]*

All participant groups were aware of the dedicated Long COVID service in HB4. However, due to the publicity the service had received and the pro-active development of a clear GP referral pathway, demand for this service quickly exceeded capacity. Staff (AHPs and Long COVID leads) expressed concern that promoting a Long COVID service in their own board may result in the same situation. Their concerns were compounded by the widely acknowledged pressure in the healthcare system due to a range of factors including staff redeployment, absence, and reopening of non-essential rehabilitation services that had been closed during previous phases of the pandemic.

> *"They* [HB4 Long COVID service] *were basically inundated. So, I had to wait, about four months or so, something like that for a slot. But it was quicker than I thought, but they were just getting overwhelmed with the workload." [P4101*]

> *"Yeah. So, it's [integrated service] not a widely publicised thing, because I don't know if we could cope as a service with the numbers." [S1102]*

For peer review only - http://bmjopen.bmj.com/site/about/guidelines.xhtml

Patients and staff reported both frustration and understanding around long waiting times for Long COVID rehabilitation:

"The referral side of it they probably done all the right things, but the timescales were just probably horrific but understandable. I know that in hindsight now so, but it didn't take away the frustrations of it." [P3208]

"Difficult due to capacity. Everywhere so busy and up against it. The pressures on us are really extreme." [L3101]

The dedicated service in HB4 was accessed by self-referral and delivered by AHP staff. Self-referral was viewed positively by patients; however, staff reported that the lack of information on self-referrals made appropriate triage of patients challenging. Health boards without self-referral and/or a dedicated Long COVID service also expressed concern that people with Long COVID would not be prioritised for rehabilitation within usual community rehabilitation services.

"I don't have self-referral at the moment. And the referrals coming through the GP or through something, some of them are coming through. But I don't know where all the others are, so, I'm not blocking access, but they are getting stuck in the system, in my system, in that if I don't have enough information on the referral, they're just going on to the normal routine waiting list." [S2207]

HB4 reported the lack of medical staff as a limitation to their dedicated service and, in keeping with other health boards, reported that the lack of a clear Long COVID pathway was a limitation, particularly as people often presented with complex issues that were unsuitable for immediate rehabilitation.

"But it's those that have the more complex needs that I wouldn't know where to send them to." [S2103]

II: Understanding Long COVID and its management

Patients commented on the burdensome nature of managing Long COVID and there being a reluctance among GPs to provide a diagnosis. They noted concerns about documenting Long COVID in healthcare records and a need to rule out other underlying conditions.

For peer review only - http://bmjopen.bmj.com/site/about/guidelines.xhtml

"It's quite difficult and just feel like, you know, you have a list as long as your arm when you actually speak to the GP because some things have changed and you know they're very sympathetic, but very clear and honest that well, we don't really know, you know? We just don't know. So, we'll give you this pill to try and treat this symptom at the moment and, that's quite hard.' [P4101]

The lack of diagnosis was also observed by AHPs and Long COVID leads, who shared a discomfort associated with labelling patients.

"What are we shaming people for having a condition? What's that all about? It's very bizarre. You get them help. I don't understand. It's like invisible illnesses." [L1202]

"It was really difficult actually to know whether to adopt the term Long COVID 'cause it's not a medical term, but we've decided in the end it's probably got wider recognition, now, that's what the patients are using. But having to be very careful, actually even when you're writing letters to GPs, you know if they didn't have confirmed COVID in the beginning, it's a presumed COVID illness, you know symptoms consistent with Long COVID, you know. It's a lot of kind of working around the houses I feel." [S4101]

Most patients expressed a need to be heard and believed by healthcare professionals. They also felt that there was powerful value in hearing that other people with Long COVID experienced similar symptoms, which provided a helpful process in validating patients' unmet needs.

"It felt to me like it was a bunch of very random symptoms. It didn't seem connected, and I think probably one of the best things in that first conversation was just hearing that the symptoms I have were very common and they are all related." [P4102]

"I get the impression that a lot of my symptoms are replicated a lot across, across other people so and I'm getting a lot of assurance on what people's progress like recovery-wise again its always pinned by keep your expectations to a minimum, like it's no [not] *going to be the miracle cure." [P3208]*

Both AHPs and Long COVID leads spoke of the challenges of understanding Long COVID and its constantly evolving evidence base, as well as the lack of data on prevalence. AHPs also reported a

For peer review only - http://bmjopen.bmj.com/site/about/guidelines.xhtml

lack of evidence to support best practice for managing Long COVID, which could result in anxiety around patient management.

> *"So, what I was concerned about was what if I give the wrong advice or the wrong sort of exercise prescription, and that I actually cause harm to him by something that I've done. And I watched some of the podcasts to sort of understand what we should and shouldn't be doing, and that is when I started to realize that there is a really strong link with Long COVID and the chronic fatigue syndrome and ME population who have been saying for years and years that, actually, sometimes, graded exercise therapy is really harmful, and you absolutely should not prescribe it." [S2104]*

To increase understanding of Long COVID, AHPs sought out peer support, online resources, and knowledge from other conditions to determine how to support people with Long COVID.

> *"There's certainly discussions around what is the best way to approach management of Long COVID and there's been a few things we can do, though, because nationally there's a huge focus on physiotherapy management of Long COVID you know, the CSP [Chartered Society of Physiotherapists], I've got lots of stuff out there, but we've also got the clinicians and the COVID rehab team there that although they're not seeing patients and more than happy to signpost people to resources and help with a bit of professional advice. I would say from a Community hub, clinician point of view, confidence hasn't been the main issue. They do seek professional advice on how to manage them." [S3203]*

III: Strengths and limitations of existing Long COVID services

A characteristic of all participating health boards during the study period was the dynamically evolving nature of the services provided. This was partly due to external factors relating to COVID prevalence, the restrictions this placed on service delivery, and financial planning decisions. Two health board areas changed their organisational structures affecting services. One health board (HB4) commenced a limited dedicated Long COVID rehabilitation service, delivered by an occupational therapist and a physiotherapist. The service developed clear pathways for referral that gained considerable publicity, as a result of which, the service was unable to meet demand. This was compounded by staff leaving their post. A considerable waiting list developed. The service ran for 18 months after which there was no further funding to continue its delivery, the service ceased and individuals on the waiting list were distributed to local community rehabilitation teams.

For peer review only - http://bmjopen.bmj.com/site/about/guidelines.xhtml

Long Covid service strengths

By Round 2 of data collection, AHPs in all health boards were able to see PwLC face-to-face, conduct home visits, have greater integration into the community, and begin working towards return-to-work packages with appropriate Long COVID patients. All these service features were perceived by clinicians to increase the flexibility of service delivery. Patients had experienced a range of modes of service delivery, largely a blended model of face-to-face individual, group, or online interventions. However, irrespective of the delivery model, most patients had a positive experience of Long COVID rehabilitation, especially the self-management information they received. PwLC reported that validation of their experience was particularly significant.

> *"I'm certainly more educated, already daily life has improved I'm not making myself ill doing things I didn't know were doing that." [P3203]*

> *"…the relief to at that point to speak to a professional who was actually acknowledging that this was happening and you know, it wasn't all in my head. I don't know there, for months and months and months I think it was common there was just no-one apart from my GP, there was just no of recognition or acknowledgement of what people were going through or anything like that. So, it was just such a relief to speak to somebody who was just genuinely interested and very supportive." [P4107]*

In the two health boards where dedicated Long COVID rehabilitation teams existed, AHPs reported that the MDT approach worked well. It was perceived to increase communication, enabling staff to develop both informal learning about Long COVID through peer discussions and also to develop their knowledge and skills in working with the emerging Long COVID clinical population through in-service learning sessions. The Long COVID leads in these areas perceived that the services were valued by people receiving their care and support, which was corroborated by the patient data.

> *"It was my GP that referred me to the psychologist then obviously the two of them were interacting so that the [COVID team] were speaking to the psychologist as well and just basically building a care package for me, so it was tailored to what I was needing physically and mentally, so they were quite good actually talking to each other as well, which was I suppose positive." [P4105]*

For peer review only - http://bmjopen.bmj.com/site/about/guidelines.xhtml

AHPs and Long COVID leads from all health boards reported high levels of staff engagement in learning about Long COVID. In the absence of specific evidence-based guidance on Long COVID rehabilitation, AHPs reported drawing on information from a wide variety of sources. Sources included a sparse literature that had been published on rehabilitation, guidance from professional bodies such as the Chartered Society of Physiotherapy and Royal College of Occupational Therapists, and learning from established approaches for conditions with similar presenting features, such as functional neurological disorder, chronic fatigue syndrome, and fibromyalgia.

AHPs and Long COVID leads reported that several staff developed an individual professional interest in the condition, worked to increase their knowledge, and sought help to learn from peers locally, across health boards in Scotland, and more widely across the United Kingdom.

> *"England has been incredibly helpful in giving and sharing lots and…you know… even within the LOCO-RISE study that's been a really helpful kind of network and relationship to support." [L3203]*

Long COVID leads in health boards where Long COVID rehabilitation services existed reported that these services enabled increased access to care with appropriate referral routes and adaptable and flexible services. One lead described the merit of a blended model of delivery and believed that telephone triage was a useful method of gaining information about patients' needs.

> *"Do you know, I think the use of the increased use of digital services has been fantastic. It's meant that we've been able to reach people when we wouldn't have normally been able to reach them. It has to be a blended model. It has to be that those people that need face-to-face intervention get it at a time when it's right. But digital advances have really made a big difference for us." [L1101]*

Long Covid service limitations

All participating health boards experienced considerable challenges in delivering Long COVID rehabilitation services. A key challenge was funding, as dedicated funding for Long COVID services had not been allocated by the Scottish Government Long COVID fund at the time these data were collected. The two health boards that had developed dedicated services used existing funding allocations. The short-term nature of this funding in one health board meant that their limited service could not be sustained, and that service ceased during Round 2 of our data collection. This

For peer review only - http://bmjopen.bmj.com/site/about/guidelines.xhtml

limited investment in developing the structure for Long COVID rehabilitation was highlighted by participants as a considerable limiting factor in service delivery, regardless of whether the health board had developed a dedicated service or not, and the *“historical underinvestment in rehab services” [L2101]* was also highlighted by long COVID leads as a significant limiting factor. Where Long COVID dedicated services had not been developed, services reported that they lacked the staffing capacity to meet the perceived additional service demand.

> *I think we're struggling even staffing wise with. We can't staff wards properly. We can't. You know all these things it's. How will we staff a Long COVID service? How will we? How? How will we best provide that service? The NHS way has always been to kind of just absorb it into existing services.” [S1202]*

However, where individuals with Long COVID were seen by existing rehabilitation services these services (described henceforth as ‘integrated services’) were observed to receive considerably fewer referrals for Long COVID rehabilitation. This appeared to be due to the reluctance in these health board areas to promote services (as discussed above). AHPs in integrated services also reported that their referral criteria were often not appropriate for people with Long COVID, resulting in referrals being rejected as inappropriate as PwLC often did not meet pre-existing service criteria for community rehabilitation. Fewer Long COVID referrals entering the services led to reduced confidence among AHPs in their ability to appropriately manage PwLC symptoms, with some admitting they lacked confidence due to Long COVID being a new and largely unknown condition for which they had no training.

> *“I suppose the other challenge from my point of view is actually just setting up a new service from scratch, so you know that's a big job in itself and setting up without the evidence base without kind of any benchmark you know to work with. And so, trying to do that alongside learning about a new disease you know with information coming out constantly.” [S4101]*

AHPs in integrated services also reported a lack of knowledge and confidence in working with people presenting with Long COVID due to the broad range of symptomatology.

> *“Hardest thing – so many different symptoms that you can have with Long COVID.” [S2204]*

For peer review only - http://bmjopen.bmj.com/site/about/guidelines.xhtml

Finally, less experienced AHPs in integrated services were reported to lack confidence in dealing with Long COVID cases as they saw fewer PwLC. This meant that in some areas increased pressure was placed on the limited capacity of senior staff.

A limitation within both integrated and dedicated rehabilitation services was the lack of medical input to both integrated and dedicated services and the challenges of onward referral to specialist secondary care.

> *"Having the right staff, having medical support in the team, that's been a big gap I feel, and that's still a gap and I think that's really important given the complexity of patients coming through. We're doing a lot of "safety netting" as AHP's at the moment, but I don't think, you know, that that's our job, necessarily. We've had to take on some of that." [S4101]*

DISCUSSION

We explored the perceptions and experiences of community rehabilitation for Long COVID from the perspectives of patients, AHPs delivering community rehabilitation services, and NHS staff leading on the Long COVID response in four Scottish health boards. We were particularly interested in exploring the barriers and facilitators to Long COVID rehabilitation experienced by these three groups across the four different health boards. We found several barriers to accessing community rehabilitation for Long COVID across both dedicated and integrated services, albeit for different reasons, and not only during periods when non-essential services were restricted. We found that the uncertainties of Long COVID, including perceived reluctance of GPs to diagnose, and the constantly evolving evidence-base were barriers to accessing rehabilitation services, and to AHPs confidently providing Long COVID rehabilitation. We also found that lack of or short-term funding, limited pre-COVID funding of community rehabilitation services, and reluctance to promote existing services to the Long COVID population were key operational barriers to Long COVID rehabilitation service provision. Facilitators included flexible services with clear referral pathways, MDT working, sourcing professional information, and support from within and outwith health board areas. While PwLC may experience frustration around accessing services and the evidence base for Long COVID rehabilitation is not firmly established, support and information received from an empathetic AHP was highly valued.

Barriers to accessing healthcare services for Long COVID have been reported in previous research focusing on GPs [11 26] and the views of people with Long COVID [27]. We have highlighted that

For peer review only - http://bmjopen.bmj.com/site/about/guidelines.xhtml

specific barriers to accessing community rehabilitation for PwLC are principally related to unclear referral pathways and demand-capacity issues. Barriers to accessing the physical rehabilitation workforce are not a new phenomenon; Long COVID has arguably brought this previously 'relatively neglected' workforce into the spotlight [28] and highlighted the need for adequate human resourcing of rehabilitation services. This concurs with Baz et al's [11] study of accessing healthcare support for Long COVID in England, which identified staff having to use existing, already stretched, services to support people with Long COVID. Brennan et al [26] reported that GPs (the principal gatekeepers to community rehabilitation services) found referral to community services challenging to negotiate. We identified a complex interplay of factors that impacted on referral from GP to community rehabilitation, including in some health boards a reluctance to promote services due to existing or anticipated capacity issues. Indeed, the dedicated Long COVID service in our study was quickly overwhelmed, demonstrating the real need for Long COVID rehabilitation, which the current epidemiology literature supports [2 29], and highlighting a capacity issue that needs to be urgently addressed.

There is currently no consensus on the optimal model of service delivery for Long COVID, beyond recommendations for it to be multilevel [14 17], multi-professional [14 30-32], person-centred [30], and adequately resourced [14]. Our findings support these recommendations. Services that are flexible to patients' needs and offer a range of delivery modes were valued by patients and staff. The benefit of multidisciplinary team working was a key theme throughout our findings, from both staff and patients' perspectives. However, the MDTs in our study were comprised of various AHPs and in some cases nurses and psychologists; access to medical specialists, as seen in some Long COVID dedicated MDT services or 'one stop' clinics [17] was notably lacking in the four participating health boards and identified as a limitation by participants.

The uncertainties around Long COVID, its diagnosis (particularly with the withdrawal of widespread testing), and optimal management have all been previously reported [33], and are inevitable given the newness of Long COVID as a condition and the as yet emerging evidence-base. It is encouraging that research on Long COVID rehabilitation is ongoing. It is vital to continue to explore Long COVID rehabilitation service delivery models, as well as the effectiveness of specific rehabilitation interventions, acknowledging that there is unlikely to be one that fits all contexts or geographical locations [14] .

Strengths and limitations

This study is the first large scale study to explore community rehabilitation services for Long COVID in Scotland. Scotland has distinct geographical and health policy landscapes that merit study of Long

For peer review only - http://bmjopen.bmj.com/site/about/guidelines.xhtml

COVID rehabilitation within its specific context. However, this is also a limiting factor as it potentially impacts the transferability of the study's findings elsewhere. However, the environment is only one of the criteria that should be examined when considering issues of transferability; the study's population and intervention characteristics should also be considered [34]. In these two respects our findings have greater transferability: PwLC present with similar health needs' internationally [2] and require similar cooperation with service providers; and while differently named, similar dedicated and integrated service models of Long COVID rehabilitation described are found elsewhere [17 35]. Ultimately it is for the reader to consider how transferable the findings of this study are to their geographical, policy and organisational context [34]. We included the perspectives of those receiving and delivering services, as well as those responsible for their oversight. Data collection took place over two rounds, enabling us to capture some of the shifting practices inevitable with a new condition. Several researchers were involved at all stages of data analysis and interpretation, including people with lived experience of Long COVID. Validity of the study findings were therefore enhanced by data (two rounds and three participant groups) and investigator triangulation (multiple researchers involved in analytical decisions) [36 37]. The study is not without limitations. We selected the four Scottish health boards based on geographical distinction and service delivery variance, as described in our 2019 national survey [13]. However, by the time we commenced data collection, approaches to Long COVID rehabilitation had already evolved meaning that there were fewer distinct service delivery differences between the four health boards. Nonetheless, our selected health board areas enabled us to investigate differences across the two principal modes of service delivery (dedicated and integrated) in Scotland at the time of writing. While we are confident we interviewed all key Long COVID leads in each participating health board and had high levels of AHP participation, thereby achieving adequate information power [25] or these two groups patient recruitment was limited by low numbers of patients accessing Long COVID rehabilitation services. This study investigated the perspectives of three key stakeholder groups: PwLC, staff delivering services and Long COVID service leads involved in wider strategic and organisational issues. Other relevant stakeholder's perspectives, such as General Practitioners/family doctors were not included in this paper but will be reported in forthcoming LOCO-RISE publications.

Implications for practice and research

There is likely to be an ongoing need for community rehabilitation to support the needs of PwLC. It is clear that a 'one-size-fits-all' model of service delivery is unlikely to be desirable in geographically diverse healthcare environments, such as that found in Scotland. The impact of geographical setting was an observably limiting factor on service delivery, as more remote communities had fewer

For peer review only - http://bmjopen.bmj.com/site/about/guidelines.xhtml

available rehabilitation staff and those that worked in remote rural communities often worked in isolation. However, the issue of rurality was confounded by included health boards with large remote rural populations also providing an integrated service delivery model of Long COVID rehabilitation. Despite this, our analysis of the data presented in this paper suggests the issue of rurality may be less pivotal than whether health boards decide to integrate Long COVID rehabilitation into existing services or develop new dedicated Long COVID rehabilitation services. Regardless of which approach is taken, this study has found several factors that require to be addressed to enable optimal Long COVID rehabilitation service delivery: proactive provision and promotion of clear referral pathways so both PwLC and primary care clinicians know of their existence and how to refer to them; sufficient staffing and range of multidisciplinary expertise to meet the broad range of rehabilitation needs that PwLC require; where Health Boards provide services in remote and rural areas, AHP staff should be enabled to access professional information and specific peer support from clinicians within and outwith their own Health Board area who have greater experience of and confidence in providing rehabilitation to PwLC. This study represents the first stage in exploring community rehabilitation for Long COVID. We have also explored the perceptions of GPs and adults with Long COVID who have tried to access rehabilitation services (to be reported elsewhere). This paper is part of LOCO-RISE, a wider realist evaluation of Long COVID rehabilitation services in the four health boards in Scotland. Future papers will present further data from this evaluation. Collectively these papers will inform contextualised, evidence-based recommendations for future Long COVID rehabilitation services in Scotland. The transferability of key findings from this Scottish-based study should be considered when considering their implications for different clinical and policy contexts.

CONCLUSION

The organisational delivery of Long COVID community rehabilitation is complex and faces several challenges. In this first of a series of outputs from our LOCO-RISE study we have provided a detailed understanding of the barriers and facilitators to Long COVID community rehabilitation from the perspectives of patients, AHPs, and Long COVID leads. The knowledge presented here can be used by those developing and delivering services for people with Long COVID. Community rehabilitation services have much to offer people with Long COVID, but their optimal delivery requires adequate planning, publicity, and resource.

Acknowledgement

We are grateful to all our participants who gave their time to take part in interviews and to the local primary investigators from the health boards; Lynne Frew, Lynn Morrison and Gail Thomson-Patel,

For peer review only - http://bmjopen.bmj.com/site/about/guidelines.xhtml

for their contributions to the LOCO-RISE study. We extend our sincere gratitude to the rehabilitation and administrative staff for supporting recruitment and data collection, and to Valery Burnett for her assistance with data collection.

Author contributions

KC, ED, JM, LA, JP, JO, AL contributed to the study's conception and design. TT, VB, JC, RM, JS undertook data collection. ED, KC, TT, JC, JS, ES, RM undertook analyses and drafted the first version of the manuscript. ED led future iterations of the manuscript. All authors read and commented on the manuscript and approved the final version of it. The corresponding author attests that all authors meet authorship criteria and that nobody meeting the criteria have been omitted. ED and KC have joint responsibility for the research conduct of the study, had access to the data, and controlled the decision to publish.

Ethics approval

Ethical approval was granted from the Wales Research Ethics Committee 6 [21/WA/0118], and each of the four health boards granted R&D management approval.

Funding statement

This work was supported by the Chief Scientist Office Scotland, grant number COV/LTE/20/29

Competing interests

All authors have completed the ICMJE uniform disclosure form at www.icmje.org/coi_disclosure.pdf and declare: all authors had financial support from the Chief Scientist Office Scotland (grant number COV/LTE/20/29) for the submitted work; ED has received research grants from NIHR and Scottish Government for the following studies: Caring for Long COVID in primary care and DBI COVID study, respectively; no other relationships or activities that could appear to have influenced the submitted work.

Data sharing

All data produced in the present study are available upon reasonable request. Robert Gordon University holds the copyright for the full interview transcripts and may grant data sharing permission on request.

REFERENCES

1. NICE SIGN RCGP. COVID-19 rapid guideline: managing the longterm effects of COVID-19 2022 [updated 28 February 2023. Available from: https://www.nice.org.uk/guidance/ng188/resources/covid19-rapid-guideline-managing-the-longterm-effects-of-covid19-pdf-510355157422022.

For peer review only - http://bmjopen.bmj.com/site/about/guidelines.xhtml

2. Davis HE, McCorkell L, Vogel JM, et al. Long COVID: major findings, mechanisms and recommendations. Nat Rev Microbiol 2023;21(3):133-46. doi: 10.1038/s41579-022-00846-2 [published Online First: 20230113]

3. Office for National Statistics (ONS). released 2 February 2023, ONS website, statistical bulletin, Prevalence of ongoing symptoms following coronavirus (COVID-19) infection in the UK 2023 [Available from: https://www.ons.gov.uk/peoplepopulationandcommunity/healthandsocialcare/conditionsanddiseases/bulletins/prevalenceofongoingsymptomsfollowingcoronaviruscovid19infectionintheuk/2february2023 accessed 28 Feburary 2023 2023.

4. NHS England. NHS sets out long COVID action plan for thousands of people with persistent symptom 2022 [Available from: https://www.england.nhs.uk/2022/07/nhs-sets-out-long-covid-action-plan-for-thousands-of-people-with-persistent-symptom/ accessed 20 July 2023 2023.

5. Welsh Government. Funding boost to open long COVID services up to people with long term conditions 2023 [Available from: https://www.gov.wales/funding-boost-open-long-covid-services-people-long-term-conditions accessed 20 July 2023 2023.

6. Scottish Government. Long COVID Support Fund 2021 [Available from: https://www.gov.scot/news/long-covid-support-fund/ accessed 20 July 2023 2023.

7. World Health Organization. Clinical management of COVID-19: living guideline, 13 January 2023. Geneva: World Health Organization, 2023.

8. DeMars J, Brown DA, Angelidis I, et al. What is Safe Long COVID Rehabilitation? J Occup Rehabil 2023;33(2):227-30. doi: 10.1007/s10926-022-10075-2

9. NHS England. National commissioning guidance for post COVID services 2022 [Available from: https://www.england.nhs.uk/publication/national-commissioning-guidance-for-post-covid-services/ accessed 28 February 2023 2023.

10. Buttery S, Philip KEJ, Williams P, et al. Patient symptoms and experience following COVID-19: results from a UK-wide survey. BMJ Open Respir Res 2021;8(1) doi: 10.1136/bmjresp-2021-001075

11. Baz SA, Fang C, Carpentieri JD, et al. 'I don't know what to do or where to go'. Experiences of accessing healthcare support from the perspectives of people living with Long Covid and healthcare professionals: A qualitative study in Bradford, UK. Health Expect 2023;26(1):542-54. doi: 10.1111/hex.13687 [published Online First: 20221213]

12. Macpherson K, Cooper K, Harbour J, et al. Experiences of living with long COVID and of accessing healthcare services: a qualitative systematic review. BMJ Open 2022;12(1):e050979. doi: 10.1136/bmjopen-2021-050979 [published Online First: 20220111]

For peer review only - http://bmjopen.bmj.com/site/about/guidelines.xhtml

13. Duncan E, Cooper K, Cowie J, et al. A national survey of community rehabilitation service provision for people with long Covid in Scotland. F1000Res 2020;9:1416. doi: 10.12688/f1000research.27894.2 [published Online First: 20201207]

14. Decary S, De Groote W, Arienti C, et al. Scoping review of rehabilitation care models for post COVID-19 condition. Bull World Health Organ 2022;100(11):676-88. doi: 10.2471/BLT.22.288105 [published Online First: 20221003]

15. Flannery T, Brady-Sawant H, Tarrant R, et al. A Mixed-Methods Evaluation of a Virtual Rehabilitation Program for Self-Management in Post-COVID-19 Syndrome (Long COVID). Int J Environ Res Public Health 2022;19(19) doi: 10.3390/ijerph191912680 [published Online First: 20221004]

16. Killingback C, Thompson M, Nettleton M, et al. Telerehabilitation for patients who have been hospitalised with covid-19: a qualitative study. Disabil Rehabil 2023:1-9. doi: 10.1080/09638288.2022.2159075 [published Online First: 20230111]

17. Parkin A, Davison J, Tarrant R, et al. A Multidisciplinary NHS COVID-19 Service to Manage Post-COVID-19 Syndrome in the Community. J Prim Care Community Health 2021;12:21501327211010994. doi: 10.1177/21501327211010994

18. Lloyd-Evans PHI, Baldwin MM, Daynes E, et al. Early experiences of the Your COVID Recovery((R)) digital programme for individuals with long COVID. BMJ Open Respir Res 2022;9(1) doi: 10.1136/bmjresp-2022-001237

19. Descriptive Phenomenological and Qualitative Description Research. West J Nurs Res 2016;38(9):1185-204. doi: 10.1177/0193945916645499 [published Online First: 20160422]

20. Tong A, Sainsbury P, Craig J. Consolidated criteria for reporting qualitative research (COREQ): a 32-item checklist for interviews and focus groups. Int J Qual Health Care 2007;19(6):349-57. doi: 10.1093/intqhc/mzm042 [published Online First: 20070914]

21. Malterud K, Siersma VD, Guassora AD. Sample Size in Qualitative Interview Studies: Guided by Information Power. Qual Health Res 2016;26(13):1753-60. doi: 10.1177/1049732315617444 [published Online First: 20160710]

22. Hoffmann TC, Glasziou PP, Boutron I, et al. Better reporting of interventions: template for intervention description and replication (TIDieR) checklist and guide. BMJ 2014;348:g1687. doi: 10.1136/bmj.g1687 [published Online First: 20140307]

23. Keith RE, Crosson JC, O'Malley AS, et al. Using the Consolidated Framework for Implementation Research (CFIR) to produce actionable findings: a rapid-cycle evaluation approach to improving implementation. Implement Sci 2017;12(1):15. doi: 10.1186/s13012-017-0550-7 [published Online First: 20170210]

For peer review only - http://bmjopen.bmj.com/site/about/guidelines.xhtml

24. Gale NK, Heath G, Cameron E, et al. Using the framework method for the analysis of qualitative data in multi-disciplinary health research. BMC Med Res Methodol 2013;13:117. doi: 10.1186/1471-2288-13-117 [published Online First: 20130918]

25. Goldsmith LJ. Using Framework Analysis in Applied Qualitative Research. 2021

26. Brennan A, Broughan J, McCombe G, et al. Enhancing the management of long COVID in general practice: a scoping review. BJGP Open 2022;6(3) doi: 10.3399/BJGPO.2021.0178 [published Online First: 20220928]

27. Kingstone T, Taylor AK, O'Donnell CA, et al. Finding the 'right' GP: a qualitative study of the experiences of people with long-COVID. BJGP Open 2020;4(5) doi: 10.3399/bjgpopen20X101143 [published Online First: 20201215]

28. Jesus TS, Landry MD, Dussault G, et al. Human resources for health (and rehabilitation): Six Rehab-Workforce Challenges for the century. Hum Resour Health 2017;15(1):8. doi: 10.1186/s12960-017-0182-7 [published Online First: 20170123]

29. Gokani SA, Ta NH, Espehana A, et al. The growing burden of long COVID in the United Kingdom: Insights from the UK Coronavirus Infection Survey. Int Forum Allergy Rhinol 2022 doi: 10.1002/alr.23103 [published Online First: 20221208]

30. Ceban F, Leber A, Jawad MY, et al. Registered clinical trials investigating treatment of long COVID: a scoping review and recommendations for research. Infect Dis (Lond) 2022;54(7):467-77. doi: 10.1080/23744235.2022.2043560 [published Online First: 20220314]

31. Nguyen NN, Hoang VT, Dao TL, et al. Clinical patterns of somatic symptoms in patients suffering from post-acute long COVID: a systematic review. Eur J Clin Microbiol Infect Dis 2022;41(4):515-45. doi: 10.1007/s10096-022-04417-4 [published Online First: 20220210]

32. Ostrowska M, Rzepka-Cholasinska A, Pietrzykowski L, et al. Effects of Multidisciplinary Rehabilitation Program in Patients with Long COVID-19: Post-COVID-19 Rehabilitation (PCR SIRIO 8) Study. J Clin Med 2023;12(2) doi: 10.3390/jcm12020420 [published Online First: 20230104]

33. O'Hare AM, Vig EK, Iwashyna TJ, et al. Complexity and Challenges of the Clinical Diagnosis and Management of Long COVID. JAMA Netw Open 2022;5(11):e2240332. doi: 10.1001/jamanetworkopen.2022.40332 [published Online First: 20221101]

34. Schloemer, T., & Schröder-Bäck, P. Criteria for evaluating transferability of health interventions: a systematic review and thematic synthesis. Implementation Science 2018;13 doi.org/10.1186/s13012-018-0751-8

35. Sivan M, Greenhalgh T, Darbyshire JL, et al. LOng COvid Multidisciplinary consortium Optimising Treatments and servIces acrOss the NHS (LOCOMOTION): protocol for a mixed-methods study in the UK. BMJ Open 2022;12:e063505. doi:10.1136/ bmjopen-2022-063505

For peer review only - http://bmjopen.bmj.com/site/about/guidelines.xhtml

36. Noble H, Heale R. Triangulation in research, with examples. Evidence-Based Nursing 2019;22:67-68. doi: 10.1136/ebnurs-2019-103145

37. O'Cathain A, Murphy E, Nicholl J. Multidisciplinary, Interdisciplinary, or Dysfunctional? Team Working in Mixed-Methods Research. Qualitative Health Research 2008;18(11):1574-85. doi: 10.1177/1049732308325535

For peer review only

For peer review only - http://bmjopen.bmj.com/site/about/guidelines.xhtml

Consolidated criteria for reporting qualitative studies (COREQ): 32-item checklist

Please indicate in which section each item has been reported in your manuscript. If you do not feel an item applies to your manuscript, please enter N/A.

For further information about the COREQ guidelines, please see Tong *et al.*, 2017: https://doi.org/10.1093/intqhc/mzm042

No.	Item	Description	Section #
**Domain 1: Research team and reflexivity**			
Personal characteristics			
1.	Interviewer/facilitator	Which author/s conducted the interview or focus group?	
2.	Credentials	What were the researcher's credentials? *E.g. PhD, MD*	
3.	Occupation	What was their occupation at the time of the study?	
4.	Gender	Was the researcher male or female?	
5.	Experience and training	What experience or training did the researcher have?	
Relationship with participants			
6.	Relationship established	Was a relationship established prior to study commencement?	
7.	Participant knowledge of the interviewer	What did the participants know about the researcher? *E.g. Personal goals, reasons for doing the research*	
8.	Interviewer characteristics	What characteristics were reported about the interviewer/facilitator? *E.g. Bias, assumptions, reasons and interests in the research topic*	
**Domain 2: Study design**			
Theoretical framework			
9.	Methodological orientation and theory	What methodological orientation was stated to underpin the study? *E.g. grounded theory, discourse analysis, ethnography, phenomenology, content analysis*	
Participant selection			
10.	Sampling	How were participants selected? *E.g. purposive, convenience, consecutive, snowball*	
11.	Method of approach	How were participants approached? *E.g. face-to-face, telephone, mail, email*	
12.	Sample size	How many participants were in the study?	
13.	Non-participation	How many people refused to participate or dropped out? What were the reasons for this?	
Setting			
14.	Setting of data collection	Where was the data collected? *E.g. home, clinic, workplace*	
15.	Presence of non-participants	Was anyone else present besides the participants and researchers?	

16.	Description of sample	What are the important characteristics of the sample? *E.g. demographic data, date*	
Data collection			
17.	Interview guide	Were questions, prompts, guides provided by the authors? Was it pilot tested?	
18.	Repeat interviews	Were repeat interviews carried out? If yes, how many?	
19.	Audio/visual recording	Did the research use audio or visual recording to collect the data?	
20.	Field notes	Were field notes made during and/or after the interview or focus group?	
21.	Duration	What was the duration of the interviews or focus group?	
22.	Data saturation	Was data saturation discussed?	
23.	Transcripts returned	Were transcripts returned to participants for comment and/or correction?	
**Domain 3: analysis and findings**			
Data analysis			
24.	Number of data coders	How many data coders coded the data?	
25.	Description of the coding tree	Did authors provide a description of the coding tree?	
26.	Derivation of themes	Were themes identified in advance or derived from the data?	
27.	Software	What software, if applicable, was used to manage the data?	
28.	Participant checking	Did participants provide feedback on the findings?	
Reporting			
29.	Quotations presented	Were participant quotations presented to illustrate the themes / findings? Was each quotation identified? *E.g. Participant number*	
30.	Data and findings consistent	Was there consistency between the data presented and the findings?	
31.	Clarity of major themes	Were major themes clearly presented in the findings?	
32.	Clarity of minor themes	Is there a description of diverse cases or discussion of minor themes?	

When submitting your manuscript via the online submission form, please upload the completed checklist as a Figure/supplementary file.

If you would like this checklist to be included alongside your article, we ask that you upload the completed checklist to an online repository and include the guideline type, name of the repository, DOI and license in the *Data availability* section of your manuscript.

Developed from: Allison Tong, Peter Sainsbury, Jonathan Craig, Consolidated criteria for reporting qualitative research (COREQ): a 32-item checklist for interviews and focus groups, International Journal for Quality in Health Care, Volume 19, Issue 6, December 2007, Pages 349–357, https://doi.org/10.1093/intqhc/mzm042

For peer review only

SUPPLEMENT File 1: Interview Topic Guides

Explanation of Supplement File 1
This file contains three interview topic guides, which were tailored for the three groups of study participants: 1) Long COVID leads; 2) Staff Delivering long COVID rehabilitation services (AHPs); and 3) People with long COVID (PwLC). The first guide, for long COVID leads, was informed by the Consolidated Framework for Implementation Research (CFIR). The CFIR categories are noted in blue.
Long COVID leads and AHPs were interviewed multiple times, whereas PwLC were interviewed at one time point. The second guide, for Staff Delivering long COVID rehabilitation, includes reframed questions in red for the second interview.

1. Interview Topic Guide for Long COVID Leads

Purpose

This topic guide supports the data collection to explore NHS Heath Board senior management perceptions of how decisions are made regarding the allocation of Scottish Government funding for Long COVID and in particular how decisions were/are made regarding funding, model of service provision and publicity for long COVID rehabilitation in community settings.

The topic guide is deliberately broad and more specific areas for questioning will be driven by the topics raised during the interview. You do not have to ask questions in the order they are presented but should use the questions as a checklist to ensure all key topic areas are covered.

SECTION 1: GENERAL INTRODUCTIONS

1.1 General introduction by interviewer to the purpose of the interview (Researcher note: relay purpose of interview as described above above).

1.2 General introduction by the interviewee of their managerial role in general and specifically in relation to long COVID.

1.2.1 Please could you tell me about your role within [name NHS Health Board]?

1.2.2 Specifically, could you tell me what role you have in the decision-making process regarding the Health Board's decision regarding delivery of long COVID? **(Process)**

1.2.3 If they do not mention their role in funding decisions relating to long COVID services probe for that.

SECTION 2: SERVICE DELIVERY

2.1 Can you describe to me how long COVID services have been provided to date by [name NHS Health Board]? (Service characteristics)

2.1.1 What are the services that are delivered for long COVID within [NHS Health Board]? (Service characteristics)

For peer review only - http://bmjopen.bmj.com/site/about/guidelines.xhtml

2.1.2 What were the drivers for introducing these services and the way in which they were/are delivered (internal {e.g. within Health Board}/external {political, societal etc})? **(Inner/Outer setting; Process)**

2.1.1 How were these services funded? **(Inner/Outer settings)**

2.1.2 Have long COVID services changed at any point within your service? [If yes, then probe regarding why they changed and in what way]. **(Service characteristics/ Inner/Outer setting)**

2.1.2 What are/were the challenges that [NHS Health Board] face(d) in delivering long COVID services? **(Inner/Outer settings; Process)**

2.3 How have your services changed (if at all) since the arrival of COVID?

2.3.1 In what way/if any/ has [NHS Health Board] promoted these services to key referrer agencies (such as GPs and others in Primary Care), and the public? What has guided the decisions you have made re promoting long COVID services to Primary Care, the community and other stakeholders?

2.3. 1 What is your general attitude towards this model of community rehabilitation? (How has it been going)? [Prompts: positive/beneficial – please elaborate, negative/detrimental – please elaborate, unclear etc.]

2.2 On 30 September 2021 the Scottish Government announced additional funding of £10 million pounds for long COVID services. How has that allocation been decided for NHS Health Boards? What share did [NHS Health Board] receive? (Inner/Outer settings; Processes)

2.2.1 Within [NHS Health Board] what was the decision-making process for how that money would be spent? **(Inner setting; Processes)**

2.2.2. Will rehabilitation services for people with long COVID in the community benefit from this funding? If yes, then how? If no, then why was spending not priorities for this? **(Intervention; Processes; Inner setting)**

2.2.3 If there will be new services, how will these new services be communicated to Primary Care and the public and other relevant stakeholders?

SECTION 3: FACILITATORS AND BARRIERS

3.1 What (if anything) do you think is working well about how long COVID services – and particularly rehabilitation for people with long COVID are being delivered for people in [NHS Health Board].

3.2.1 What has enabled this/these to work well?

3.2 What (if any) barriers do you feel there are in delivering high quality services for people with long COVID, in particular that requiring long COVID rehabilitation? How might these barriers be overcome?

For peer review only - http://bmjopen.bmj.com/site/about/guidelines.xhtml

SECTION 4: THE FUTURE

4.2 How do you see long COVID services being delivered over the coming months and years?
(Intervention; Outer/Inner Setting; Process)

4.1 How do you foresee long COVID rehabilitation in [NHS Health Board] being delivered over the coming months and years?

4.1.1 What are the funding implications?

SECTION 5: CLOSING THE INTERVIEW

5.1 Is there anything else we have not covered regarding funding and delivery of long COVID, particularly in relation to rehabilitation in the community?

Notes for interviewer

- Check topic guide and return to any questions as necessary
- If no further questions/discussion remains, thank participant for their time
- Save/stop the recording
- Note down any interviewer/participant observations
- Upload recording to CSO R Drive (RGU) and include any notes taken

For peer review only - http://bmjopen.bmj.com/site/about/guidelines.xhtml

2. Interview Topic Guide for Staff Delivering Long COVID Rehabilitation Services (AHPs)

Aim

To explore staff perceptions of how community rehabilitation for people experiencing symptoms of long COVID is being delivered. To explore their perception of the barriers (and how these could be overcome) and facilitators (and how these could be optimised) for success. To explore their perception of the impact of community rehabilitation on patient outcomes for this population. The interviews have a general purpose to obtain feedback from those involved in delivering community rehabilitation for people with long COVID.

The specific aim of follow-up interviews is to get a sense of what, if anything has changed in the last few months. This could be at an organisational level, or at an individual professional level in relation to long COVID.

Remember – we want you to have a good conversation with people about community rehabilitation for people with long COVID. The questions are there to provide a bit of structure and to make sure you cover all required basis, but it is not an interview schedule and you do not have to follow them in order.

Tasks at or before start of interview:

(Re)Introduce self and relax participant through welcoming conversation

Check participant has read participant information sheet and understood aims and consents

Assign code to each new participant (e.g. L001 for Lanarkshire participant). Use previous code if a repeat interview assigning the letter b (if 2nd interview).

Participant information to be gathered at start of each interview: profession, gender, years qualified, Agenda for Change band (Ask if what 'Band' they are; this is their professional grade), length of time they have worked in community rehabilitation. Check that details have stayed the same since last interview if it is a repeat interview. If not, note new details.

SECTION 1: GETTING STARTED

Can you describe your role within the community rehabilitation team?

How has this role been for the last few months? Have there been any changes in relation to long COVID patients?

In this role, have you had any seen any/many patients who are experiencing long COVID?

> *(if they have seen long COVID patients)* Can you tell me about what your role has been with patients with long COVID? Has this changed at all over the last few months?
>
> *(if they have seen long COVID patients)* How confident have you been in delivering therapy for people with long COVID symptoms? Has this changed at all over the last few months?

For peer review only - http://bmjopen.bmj.com/site/about/guidelines.xhtml

(if not seen any) Have you been aware of any of your colleagues treating people due to problems related to long COVID? What have they done? Has this changed at all over the last few months?

SECTION 2: SERVICE DELIVERY

(Covers TiDIER criteria – Why, What Who, How, Where, When & How much, Tailoring)

Tell me about rehabilitation for patients with long COVID within your team/department?

Have there been any changes to how long COVID is delivered in your service? If so, can you describe these changes?

- How are people referred to your service? Who by? How do you let people know about your service?
- Why are people being referred for long COVID rehabilitation?
  - What problems? What are the general goals of rehabilitation for the people that have been seen?
- What happens after you received a referral (triage? Who decides who sees the person? How is initial contact (telephone/online/f2f)
- What happens after initial contact?
- What rehab services are offered?
  - e.g. assessment & advice only/self-management & signposting/set number of sessions/individual to patient needs.
  - How is that process decided?
  - Who provides rehabilitation? How is it decided who sees each patient?
- Where forms of therapy are generally delivered?
  - what do the professionals do?
- Typically, how intense is the rehabilitation process?
  - frequency/duration of interventions, how long on caseload?
- Is this different or the same as other patients accessing your service/department?
- In what way is the therapy tailored to the individual being treated?
- How do you decide when to stop intervention?

SECTION 3: FACILITATORS AND BARRIERS

What is your perception of the potential of community rehabilitation for people with long COVID?

Has this changed at all over the last few months?

- What seems to work well for people who have long COVID? (Consider: engagement, outcomes, access to services, development of service etc.)
  - Why/in what way?

For peer review only - http://bmjopen.bmj.com/site/about/guidelines.xhtml

- What doesn't work so well in delivering community rehabilitation for people with long COVID (Consider: referral process, mode of therapy delivery, duration of intervention, admin, resources etc.)
    - Why/how could these be overcome?

SECTION 4: THE FUTURE

How could services change to better suit needs of patients with long COVID who need rehabilitation?

What do you see as the big challenges facing long COVID rehabilitation just now?

- Consider: staffing: funding, mode of delivery, any services not currently provided, referral to other services, central long COVID clinics vs integrated services within existing structures.

SECTION 5: CLOSING THE INTERVIEW

Is there anything else we have not talked about but you think would be beneficial in our understanding of community rehabilitation for people with long COVID?

Notes for interviewer:

- Check topic guide that all key areas are covered
- Thank participant for their time
- Save and stop recording
- Note down any participant observations or thoughts re the interview
- Upload recording to CSOR Drive (RGU) with any notes taken

For peer review only - http://bmjopen.bmj.com/site/about/guidelines.xhtml

3. Interview Guide for People with Long COVID (PwLC)

Aim

To obtain input from people living with long COVID, their perceptions of the acceptability of the community rehabilitation service, their preferences for service delivery models and what a responsive service might look like (referral, location, duration, modes of delivery etc…).

Interviews have a general purpose to obtain ideas from participants about what an ideal community rehabilitation service for people living with long COVID might look like, what they perceive to be the barriers and facilitators to the model they are experiencing and what gaps, if any, they feel exist in service delivery. Therefore, the topic guide is deliberately broad and more specific areas for questioning will be driven by the questions and interests of the people taking part.

For peer review only

Welcome and introductions.

Check they can hear ok.

Thank you for agreeing to meet with me. Introduce self to participant.

I hopefully won't take too much your time up.

Structure of call.

Could I just start by going though the verbal consent, just to make sure that you are happy with everything? Inform them that we will go through the consent process which asks a few statements and if you can say yes/no that would be great. Just so you know that the interview will be audio recorded just so we can refer back to the data if that's ok with you.

I have 8 statements that I will read and if you are happy, if you can just say yes at the end of them that would be great.

Ask to say name. <CONSENT>

Great, thank you for doing that – it just keeps us all right. I will stop the recording there.

Opportunities for the participant to ask questions about the study and to confirm verbally that they are still happy to participate.

Re-start new recording

Opening statement:

Thank you again for taking the time to speak to me. And just to give you a bit of background about the study. Essentially, we're doing a project over two years just looking at long COVID rehabilitation across Scotland. We're looking across four Scottish health boards. And as you can understand we are still learning about long COVID and things are changing very quickly. So, we are kind of trying to see how things are changing over these two years right from start in relations to rehabilitation service. What we've been doing is speaking to staff that deliver the service across Scotland also sort of managers and directors at each of those health boards. But we also want to hear from people like yourselves and who have experienced COVID and experiencing long COVID, just in terms of getting

For peer review only - http://bmjopen.bmj.com/site/about/guidelines.xhtml

your viewpoints about your experiences, what's been good, what's been bad and so forth. We are really interested to hear things from your side of things if that's alright?

Start Interview questions

These can be asked in any order and are there to guide discussion. Feel free to use your own words to ask the questions in as open a manner as possible. What is important is the topics are covered.

Q1. Can you tell me a bit about your COVID story?

Prompts: When were you diagnosed? Was it a formal diagnosis following a test or did you feel you matched the symptoms? When did you begin to consider you had long COVID? How is COVID affecting you now?

Prompts:
Job: What is your job now?
Symptoms mentioned? Any other symptoms?
Did you seek any help from GP? What did the GP do? Refer to other services? What services?
And how did that work for you?
Has that changed? Have you had any face-to-face appointments or is it just stayed virtual, online?
Anything you thought could be better? What do you think went well for you?

Q2. How did you get referred for long COVID rehabilitation?

Prompts: Who referred you?
What did they refer you for (e.g. fatigue, breathlessness, brain-fog etc.),
was it easy to get referred?
How long did they wait to be seen?

Q3. For how long and how often have you been receiving long COVID rehabilitation (weeks and sessions [**when and how much]**)?

Q4. What had you hoped/do you hope to achieve from community rehabilitation?

Q5. Can you tell me a bit about the rehabilitation you have received to date?

Prompts: **Who** has provided your long COVID rehab (i.e. what type of professionals – physiotherapists, occupational therapists etc);
What has your rehabilitation consisted of so far (i.e. what procedures or advice, or other activities); **How** was it provided? (e.g. face to face or in a group, online or telephone);
Where was it provided? (if face to face was it at home or in a clinic etc)

Q6. What are your experiences of long COVID rehabilitation to date?

Prompts: Do you feel the service is/was responsive to your needs?
How do you feel about the number of times and frequency with which you were seen?
How appropriate do you think the service's way of delivering rehabilitation has been/is (e.g. face to face/digital/self-management) for you?

Q7. Are there any particular aspects that you think are working well or lessons learned to improve long COVID rehabilitation you are receiving?

Prompt: What is working well?
What could be better? (Note – try and tap into service issues such as referral, delivery mode etc, as well as individual issues)

For peer review only - http://bmjopen.bmj.com/site/about/guidelines.xhtml

Q8. Is there anything else you would like to share about your experience of the community rehabilitation service?

Closing statement:

That's all the questions I've got. Is there anything else that we've not touched on that you think you know important to mention.

Great. Well, thank you again. Lovely to meet you and thank you. As you said, you're happy enough if there's anything else in the future, if you're happy for me to get back in touch.

I was going to ask if there's anything in the future that I think of, would you be happy for me to contact you again just via email or something and see how you're getting on?

Thanks, and conclude interview.

Turn off recorder

For peer review only

For peer review only - http://bmjopen.bmj.com/site/about/guidelines.xhtml
